# Antibiotic Production and Antibiotic Resistance: The Two Sides of AbrB1/B2, a Two-Component System of *Streptomyces coelicolor*

**DOI:** 10.3389/fmicb.2020.587750

**Published:** 2020-10-09

**Authors:** Ricardo Sánchez de la Nieta, Sergio Antoraz, Juan F. Alzate, Ramón I. Santamaría, Margarita Díaz

**Affiliations:** ^1^Instituto de Biología Funcional y Genómica/Departamento de Microbiología y Genética, Consejo Superior de Investigaciones Científicas/Universidad de Salamanca, Salamanca, Spain; ^2^Departamento de Microbiología y Parasitología, Facultad de Medicina, Centro Nacional de Secuenciación Genómica, Sede de Investigación Universitaria, Universidad de Antioquia, Medellín, Colombia

**Keywords:** two-component system, *Streptomyces*, antibiotic production, antibiotic resistance, vancomycin, RNA-Seq

## Abstract

Antibiotic resistance currently presents one of the biggest threats to humans. The development and implementation of strategies against the spread of superbugs is a priority for public health. In addition to raising social awareness, approaches such as the discovery of new antibiotic molecules and the elucidation of resistance mechanisms are common measures. Accordingly, the two-component system (TCS) of *Streptomyces coelicolor* AbrB1/B2, offer amenable ways to study both antibiotic production and resistance. Global transcriptomic comparisons between the wild-type strain *S. coelicolor* M145 and the mutant Δ*abrB*, using RNA-Seq, showed that the AbrB1/B2 TCS is implicated in the regulation of different biological processes associated with stress responses, primary and secondary metabolism, and development and differentiation. The Δ*abrB* mutant showed the up-regulation of antibiotic biosynthetic gene clusters and the down-regulation of the vancomycin resistance gene cluster, according to the phenotypic observations of increased antibiotic production of actinorhodin and undecylprodigiosin, and greater susceptibility to vancomycin. The role of AbrB1/B2 in vancomycin resistance has also been shown by an *in silico* analysis, which strongly indicates that AbrB1/B2 is a homolog of VraR/S from *Staphylococcus aureus* and LiaR/S from *Enterococcus faecium*/*Enterococcus faecalis*, both of which are implied in vancomycin resistance in these pathogenic organisms that present a serious threat to public health. The results obtained are interesting from a biotechnological perspective since, on one hand, this TCS is a negative regulator of antibiotic production and its high degree of conservation throughout *Streptomyces* spp. makes it a valuable tool for improving antibiotic production and the discovery of cryptic metabolites with antibiotic action. On the other hand, AbrB1/B2 contributes to vancomycin resistance and is a homolog of VraR/S and LiaR/S, important regulators in clinically relevant antibiotic-resistant bacteria. Therefore, the study of AbrB1/B2 could provide new insight into the mechanism of this type of resistance.

## Introduction

The emergence and spread of antibiotic resistant bacteria currently present great challenges to humans as highlighted by the World Health Organization (WHO) and the American and European Centers for Disease Control and Prevention, CDC and ECDC, respectively. Antibiotic resistance can affect any person at any time of life and poses many risks, especially with respect to advancements in modern health care such as organ transplants and cancer therapy. The latest global antimicrobial resistance and use surveillance system (GLASS) report published by the WHO stated that over two million people are infected with antibiotic resistant bacteria (published in 2020, data collected until July 2019). However, if we consider the lack of data from countries like China (data only submitted from 78 countries/territories), and the partial implementation of surveillance systems in the countries enrolled in GLASS, the magnitude of the problem could even be considered substantially worse than the data suggests. The negative impact of this threat not only affects human health, but also animal health and world economies (CDC, 2019; GLASS, 2020).

The development and implementation of strategies against the spread of superbugs is a priority. Although raising social awareness and preventative measures can be effective, the discovery of new antibiotics that improve patient treatment options and outcomes still remain essential. In relation to this, the ubiquitous genus *Streptomyces*, with more than 600 species and several sequenced genomes, has a great capacity to produce secondary metabolites with a myriad of antimicrobial, antitumoral, and antifungal activities. Many of these metabolites are considered cryptic, owing to the lack of or the reduced ability to be produced in laboratory conditions (Hwang et al., 2014; Barka et al., 2016; Gómez-Escribano et al., 2016; Blaskovich et al., 2017; van der Meij et al., 2017; van der Heul et al., 2018; Mouncey et al., 2019).

The use of two-component systems (TCSs) are among the several strategies used to fight antibiotic resistance and are global regulators that coordinate biological processes in bacteria in response to internal and external stimuli. Many TCSs are involved in the regulation of resistance mechanisms through the activation of antibiotic response systems and changes in cell physiology that increase antibiotic resistance, making them a promising therapeutic target (Bhagirath et al., 2019; Tierney and Rather, 2019). For example, GraR/S is involved in resistance to cationic antimicrobial peptides in *Staphylococcus aureus* through the modification of teichoic acids that changes the bacterial surface charge (Yang et al., 2012), or CopR/S and CzcR/S which increase carbapenem resistance through the repression of a porin in *Pseudomonas aeruginosa* (Perron et al., 2004; Caille et al., 2007; Raavi et al., 2017). In addition, TCSs are also involved in the control of antibiotic production and have been extensively used in yield enhancement and metabolite mining (Liu et al., 2013; Rodríguez et al., 2013; Baral et al., 2018; Xia et al., 2020). For instance, MtrA/B acts as a positive regulator of chloramphenicol biosynthesis in *Streptomyces venezuelae* (Som et al., 2017) and PhoR/P is implied in the control of antimicrobial production at least in three *Streptomyces* species (Santos-Beneit, 2015): *Streptomyces lividans* (Sola-Landa et al., 2003), *Streptomyces coelicolor* (Santos-Beneit et al., 2009; Fernández-Martínez et al., 2012), and *Streptomyces natalensis* (Mendes et al., 2007).

Canonical TCSs are composed of a histidine kinase (HK) and a response regulator (RR). HK detects changes outside or inside the cells and, through the activation of the corresponding RR, trigger cellular responses, usually by regulating gene expression. This regulation is based on a histidine-aspartic phosphorylation cascade from the HK to the RR. The number of TCSs in an organism is dependent on ecologic niche, and members of the genus *Streptomyces* have approximately 90 TCSs (Álvarez et al., 2016).

In *S. coelicolor*, 23 out of the 100 TCSs in its genome have been studied, of which many have been linked to antibiotic regulation (Rodríguez et al., 2013). Some of these TCSs are global regulators of several antibiotic pathways, such as AbsA1/A2 (Lewis et al., 2019), DraK/R (Yeo et al., 2014), and RapA1/A2 (Lu et al., 2007), while others only regulate single secondary metabolite gene clusters such as CutR/S (Chang et al., 1996) and EcrA1/A2 (Li et al., 2004).

In our laboratory, the search for TCSs in *S. coelicolor* implicated in antibiotic production has led to the identification of three systems partially similar to the antibiotic regulatory system AbsA1/A2 and a related orphan RR. These systems are: AbrA1/A2 (*SCO1744/45*), AbrB1/B2 (*SCO2165/66*), AbrC1/C2/C3 (*SCO4598/97/96*), and Aor1 (*SCO2281*). The AbrA1/A2 system plays a negative role in both antibiotic production and differentiation (Yepes et al., 2011; Rico et al., 2014b). The AbrC1/C2/C3 system, an atypical TCS with two HK and one RR, acts as a positive regulator of antibiotic production and differentiation (Yepes et al., 2011; Rico et al., 2014a; Rodríguez et al., 2015). And Aor1 seems to be crucial in the antibiotic production linked to stress responses (Antoraz et al., 2017). Moreover, the biotechnological application of these systems has been demonstrated, where the overexpression of the RR AbrC3 in different species of *Streptomyces* enhanced antibiotic and antitumoral production (Rico et al., 2014a; Becerril et al., 2018). The use of a deletion mutant of the negative regulator system AbrA1/A2, as a heterologous host for antibiotic biosynthetic gene clusters, has also been successful (Rico et al., 2014b).

In this work we describe the role of AbrB1/B2 in *S. coelicolor*. The phenotypic and transcriptomic analyses of this TCS show that AbrB1/B2 is a negative regulator of antibiotic production and a positive regulator of vancomycin resistance. Also, an *in silico* analysis indicates that this system is a homolog of VraR/S and LiaR/S from *S. aureus* and *Enterococcus faecium*/*Enterococcus faecalis*, respectively, which plays an important role in the mechanism of vancomycin resistance in these pathogenic organisms (Kuroda et al., 2000; Qureshi et al., 2014; Tierney and Rather, 2019).

## Materials and Methods

### Strains, Media, and Growth Conditions

Information on the bacterial strains is shown in Supplementary Table 1. *Escherichia coli* DH5α and ET12567 (non-methylating strain) were cultivated at 37°C in LB medium and were used to manipulate DNA and to transform *S. coelicolor* respectively (this organism does no admit methylated DNA). *S. coelicolor* strains were cultivated at 30°C, R2YE was used for the transformations, MSA for sporulation (Kieser et al., 2000) and YEPD (Rose et al., 1990) for spore quantification.

For the phenotypic assay on solid media, *S. coelicolor* strains were grown on NMMP, YEPD, NA, LB, R2YE, and R5. The phenotypic assay as well as the cultures for the remaining experiments were performed using NMMP liquid media.

In the antagonistic assay, patches of *S. coelicolor* strains were grown during 10 days at 30°C on NMMP plates. Then, circular sections of the patches were collected and placed on BHI plates inoculated with a lawn of *Staphylococcus epidermidis* or *E. faecalis*. The plates were first incubated at 4°C for 5 h (to allow diffusion of any produced molecules) and then at 37°C for 24 h.

When necessary, the media were supplemented with the following antibiotics: *E. coli* media – ampicillin (100 μg mL^–1^), apramycin (15 μg mL^–1^), kanamycin (50 μg mL^–1^), chloramphenicol (25 μg mL^–1^), or nalidixic acid (25 μg mL^–1^), and *S. coelicolor* media – neomycin (20 μg mL^–1^), or thiostrepton (10 μg mL^–1^).

Photographs of colonies were obtained using an *OLYMPUS E-620* camera. Photomicrographs were taken with a *RT Monochrome* (SPOT) in an *Axiophot* (ZEISS) microscope.

### DNA Manipulation and Plasmid Construction

The plasmids and primers used in this work are listed in Supplementary Tables 2, 3, respectively. Plasmid isolations, restriction enzyme digests, ligations and *E. coli* and *S. coelicolor* transformations were carried out using the methods by Green and Sambrook (2012) and Kieser et al. (2000), following the manufacturer’s instructions.

The complementation plasmid pKCAbrB was generated in several steps. The DNA fragment including the *abrB1*/*B2* operon and its promoter was amplified from the *S. coelicolor* M145 genome by PCR using DSM-002 and DSM-004 primers and cloned into the *Eco*RI/*Hin*dIII sites of pXHis1 to yield pXAbrB. Afterward, the *Bgl*II fragment from this plasmid containing the genes and promoter was cloned into the same site of pKC796.

The multicopy plasmid pNBAbrB was generated by cloning the DSM-002/DSM-004 DNA fragment previously indicated into the *Eco*RI/*Hin*dIII sites of pN702GEM3.

### Mutant Construction

The pCRISPR-Cas9 plasmid provided by Tong et al. (2015) was used to generate the tools needed for creating the AbrB1/B2 system deletion mutant. The single guide RNA (sgRNA) used to target *abrB1/B2* genes (GCAGGACACGGATCGTCATA) was designed using the web resource http://staff.biosustain.dtu.dk/laeb/crispy_scoeli/ and the whole sgRNA guide was amplified by PCR using primers SAM-051 and SAM-067. pCRISPR-Cas9 was used as template and the amplified product was cut with *Nco*I/*Sna*BI enzymes and introduced into pCRISPR-Cas9 digested with the same enzymes, yielding the pCRISPR-Cas9-sgB plasmid. In parallel, a DNA fragment containing the flanking regions of the AbrB1/B2 system was introduced into this new plasmid to provide a homologous recombination template to repair the cut produced by Cas9 and to delete the *abrB1/B2* operon in *Streptomyces*. First, two PCRs were performed to generate fragments containing either the 1-Kb upstream *abrB1/B2* operon (oligonucleotides SAM-069/SAM-070) or the 1-Kb downstream operon (oligonucleotides SAM-071/SAM-072). Afterward, the final DNA fragment used as a template was obtained by an overlapping PCR using primers SAM-069/SAM-072. This fragment was introduced into the *Xba*I site of pCRISPR-Cas9-sgB plasmid to generate the final plasmid pCRISPR-Cas9-AbrB. All of the resulting plasmids were sequenced for verification.

All the three plasmids, the empty one (pCRISPR-Cas9), the one harboring only the sgRNA (pCRISPR-Cas9-sgB) and the final plasmid with the guide and the template (pCRISPR-Cas9-AbrB), were introduced into *E. coli* ET12567/pUZ8002 by transformation, and subsequently into *S. coelicolor* by interspecific conjugation; apramycin-resistant colonies were then selected. To induce the loss of the plasmids (which were thermosensitive), the colonies obtained were grown at 37°C for 48 h in R2YE medium; apramycin-sensitive colonies were then selected. Nalidixic acid was used to eliminate any remaining *E. coli* colonies.

Genomic DNA was extracted from the putative *S. coelicolor* M145 wild-type strain and the corresponding Δ*abrB* mutant, and the correct deletion of *abrB1/B2* genes was verified by PCR (external primers EP: RCD-005/RCD-006; and internal primers IP: RCD-007/RCD-008) (Supplementary Figure 1).

### Antibiotic Production Analysis

Antibiotic production was assayed on different solid media plates inoculated with 10^5^ spores added in a 5 μL drop, incubated for several days at 30°C, and monitored for any changes in phenotype (two drops of each strain per plate). RED production is detected as red-colored colonies and ACT production as a blue halo around the colonies.

Antibiotic production was quantified from cultures in liquid NMMP medium inoculated with 4 × 10^6^ spores mL^–1^. ACT and RED antibiotic production were quantified using the spectrophotometric method described in Yepes et al. (2011).

These experiments were all performed in quadruplicate.

### Comparative RNA-Seq Analysis

*Streptomyces coelicolor* M145 wild-type strain and its mutant Δ*abrB* were grown in liquid NMMP medium inoculated with 4 × 10^6^ spores mL^–1^ at 30°C for 24 and 36 h in triplicate biological samples. The cells were collected and treated with *RNAprotect Bacterial Reagent* (QIAGEN), and RNA was extracted using the *RNeasy Mini Kit* (QIAGEN) and treated with *RNase-free DNaseI* (Thermo Fisher Scientific). The quantity and quality of the RNA samples were checked using a bioanalyzer *2200 TapeStation* (Agilent).

Macrogen Inc. (South Korea), provided the RNA-Seq data. The rRNA was removed using the *Ribo-Zero rRNA Removal Kit* and one library per sample was obtained using *TruSeq Stranded Total RNA* (NEB Microbe). Sequencing was performed in a *NovaSeq6000* (Illumina), generating PE (paired-end) reads of 150 bases. The sequencing data were converted into FASTQ raw data for the RNA-Seq analysis using Illumina package *bcl2fastq*.

*RaNA-Seq* software was used to perform the FASTQ pre-processing (*fastp 0.19.4*) and quantification (*salmon 0.9.1*) of the samples (Prieto and Barrios, 2019). Total reads obtained for each library ranged between 36 and 52 million, and around 99% passed the quality filter; between 89 and 94% of the cleaned-read set was successfully mapped to the reference genome (*Streptomyces coelicolor* A3(2) chromosome; GenBank NC_003888). The comparative analysis was performed with *DEseq2* (Wald’s test and parametric fit type applied). STRING-Database^1^ was used for the detailed study of the results obtained.

The RNA-Seq data have been deposited in the Sequence Read Archives (*BioProject ID*: PRJNA637401; *BioSample Accessions*: SAMN15105054, SAMN15105055, SAMN15105056, SAMN15105057).

### Quantitative Reverse Transcription PCR

Oligonucleotides were designed using the *Primer Quest Tool* (IDT) (Supplementary Table 3). These experiments were performed in quadruplicate. RNA was obtained using the *RNeasy Mini Kit* (QIAGEN) and treated with *RNase-free DNaseI* (Thermo Fisher Scientific). cDNA was synthesized using *iScript Reverse Transcription Supermix* (Bio-Rad). Quantitative reverse transcription PCR (RT-qPCR) was carried out using the *TB Green^TM^ Premix Ex Taq^TM^* (TAKARA). The manufacturer’s instructions were followed in all of the previous steps. Technical triplicates were performed for each sample and the RT and NTC controls were included to check the absence of DNA or environmental contamination. The control *rpsL* (*SCO4659*) was used as the housekeeping gene. The amplification profile was monitored on a *CFX96 Touch Real Time PCR Detection System* (Bio-Rad). Absolute quantification was performed using decimal serial dilutions of *S. coelicolor* genomic DNA with a known number of copies as the standard reference.

### Assays for Testing Vancomycin and Penicillin G Resistances

Plates containing NMMP solid medium were inoculated with 2 × 10^5^ spores (and the serial dilutions) of the *S. coelicolor* M145 wild-type strain and the deletion mutant Δ*abrB*. Increasing vancomycin or penicillin G concentrations were used (1, 10, 50, 100, and 500 μg mL^–1^) to test the susceptibility of the strains to these two antibiotics. Survival rates were calculated as the number of growing colonies at each antibiotic concentration in comparison to the number of colonies growing on NMMP without it. The experiments were performed in quadruplicate.

### Sequences Pairwise Alignments

Comparison of amino acid sequences were performed in *EMBOSS Needle* (EMBL-EBI), which creates an optimal global alignment of two sequences using the Needleman–Wunsch algorithm, applying a BLOSUM62 substitution matrix (Madeira et al., 2019).

### Maximum Likelihood Analysis

The relation among the RRs was inferred using the maximum likelihood method and the Le Gascuel model (Le and Gascuel, 2008). The tree with the highest log likelihood (−2758.14) is shown in Supplementary Figure 2. The percentage of replicate trees in which the associated taxa clustered together in the bootstrap test (1000 replicates) is shown next to the branches. The initial tree for the heuristic search was obtained automatically by applying the neighbor-join and BioNJ algorithms to a matrix of pairwise distances estimated using the JTT model and then by selecting the topology with superior log likelihood value. A discrete Gamma distribution was used to model the evolutionary rate differences among sites (five categories, +*G* parameter = 21.3358). The tree is drawn to scale, with branch lengths measured in the number of substitutions per site. This analysis involved five amino acid sequences. There was a total of 242 positions in the final dataset. The analysis was conducted in *MEGA X* (Kumar et al., 2018).

### Homology Modeling

The homology modeling of the AbrB1 RR was performed in *SWISS-MODEL* (Waterhouse et al., 2018) using the VraR structure (4GVP.1.A) as a template. The model obtained shows a GMQE of 0.69 and a QMEAN of −1.08. High local quality was obtained in the REC domain and in the HTH-LuxR domain, while the local quality of the linker region was lower due to the presence of gaps. The structure assessment of the model was performed using *MolProbity v4.4* (Chen et al., 2010) and the score obtained was 1.17.

## Results

### AbrB1/B2 Is a Canonical Two-Component System Highly Conserved in Streptomycetes

AbrB1/B2 is a canonical TCS comprised by a RR (*SCO2165*, named as *abrB1*) and a HK (*SCO2166*, named as *abrB2*) coupled in the genome (Supplementary Figure 3A). Both genes seem to form an operon and their coding regions overlap by four nucleotides.

The RR AbrB1 belongs to the NarL family which is characterized by a REC receiver domain (position 3–119; contains the putative phosphorylation site: Asp55) and an HTH-LuxR DNA-binding domain (position 162–218) (Supplementary Figure 3B). The HK AbrB2 shows a classic architecture domain, containing three putative transmembrane regions (positions 21–36, 46–68, and 120–142), an extracellular input domain (position 69–119), a phosphoacceptor/dimerization HiskA-3 domain (position 193–261; contains the putative phosphorylation site: His203), and a HATPase-c domain (position 286–399) (Supplementary Figure 3C) [SMART database^2^ (Letunic and Bork, 2018), P2CS database^3^ (Barakat et al., 2011)].

AbrB1/B2 is highly conserved in *Streptomyces* spp. This TCS is present with a high degree of amino acid sequence identity (over 75% in the case of the RR) to a wide range of *Streptomyces* species: *Streptomyces griseus*, *Streptomyces scabiei*, *Streptomyces venezuelae*, *Streptomyces avermitilis*, *Streptomyces albus*, and *Streptomyces hygroscopicus* among others (Supplementary Figure 4).

### AbrB1/B2 Is a Negative Regulator of Antibiotic Production

The complete *abrB1/B2* TCS was deleted in *S. coelicolor* M145 using the CRISPR-Cas9 system (Tong et al., 2015; Supplementary Figure 1).

**FIGURE 1:**
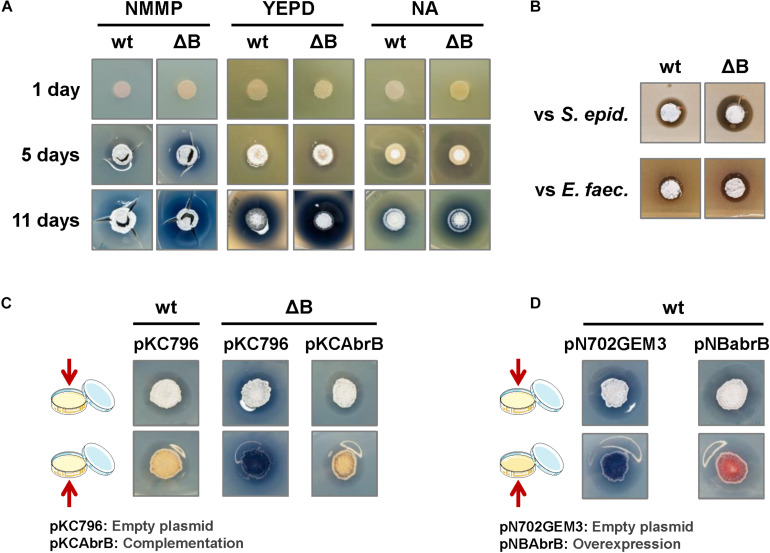
Phenotypic analysis of the *S. coelicolor* mutant Δ*abrB* strain. **(A)** Comparison of ACT production (blue halo around the colonies) and the differentiation of *S. coelicolor* M145 wild-type strain (wt) and its deletion mutant Δ*abrB* (ΔB) growing during 1, 5, and 11 days on several types of media (NMMP, YEPD, and NA). Photos of the top view of the plates. **(B)** Antagonistic assay: inhibitory effect of *S. coelicolor* M145 wild-type strain (wt) and its mutant Δ*abrB* (ΔB) growing on a lawn of the Gram+ bacteria *Staphylococcus epidermidis* (*S. epid.*) and *Enterococcus faecalis* (*E. faec.*). Photos of the top view of the plates. **(C)** Complementation of ACT production in *S. coelicolor* M145 Δ*abrB* mutant (ΔB) with the integrative plasmid pKCAbrB after 5 days on NMMP medium. The wt and ΔB strains carrying the empty plasmid (pKC796) were used as controls to compare the phenotypes. Photos of the top and bottom views of the plates. **(D)** Overexpression of the AbrB1/B2 system (pNBabrB) compared with the empty plasmid (pN703GEM3), used as control, in the wild-type strain (wt) on NMMP medium after 10 days. Photos of the top and bottom views of the plates.

The phenotype of the deletion strain, Δ*abrB*, was analyzed on several solid culture media: NMMP, YEPD, NA, LB, R2YE and R5. There were no observable differences related to morphological differentiation (appearance of aerial mycelium and sporulation) between the wild-type and mutant strains on any of the media tested. Nevertheless, enhanced actinorhodin (ACT) production was observed as a more intense or wider blue halo around the colonies on NMMP, YEPD, and NA media (Figure 1A). According to these observations, a wider inhibitory halo was produced in the mutant strain, compared to the wild-type, when the Gram+ bacteria *S. epidermidis* or *E. faecalis* were added to the media (actinorhodin is an antibiotic active against Gram+ bacteria) (Figure 1B). We chose to use the minimal medium NMMP for further assays.

The integrative plasmid pKCAbrB carrying the *abrB1/B2* system under its own promoter was able to complement the Δ*abrB* phenotype, abolishing the increment of ACT production observed in the deletion mutant (Figure 1C). On the contrary, the overexpression of the *abrB1/B2* system with the multicopy plasmid pNBAbrB (*abrB* genes under the control of its own promoter) produced a phenotype where *S. coelicolor* M145 exhibited reduced antibiotic production (Figure 1D).

**FIGURE 2:**
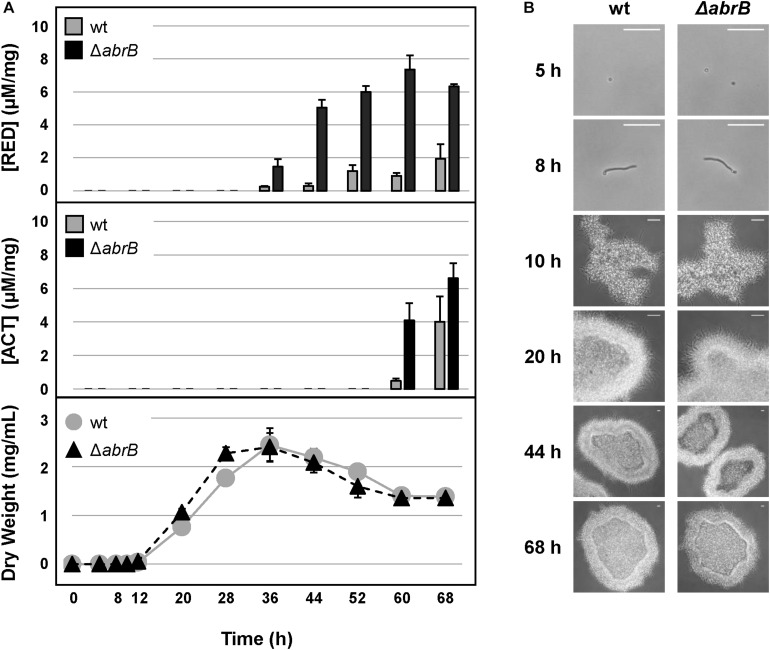
Antibiotic production and growth in liquid medium. **(A)** RED (top) and ACT (middle) production (μM/mg mycelium) along the growth curves of *S. coelicolor* M145 wild-type strain (wt, gray circles) and the mutant Δ*abrB* (black triangles) in NMMP liquid medium (bottom). Error bars show the standard deviation of the quadruplicate assays. **(B)** Germination and growth progression of the wt and Δ*abrB* strains (photomicrographs). White-bar (up-right): 50 μm.

The increment in antibiotic production was also observed in NMMP liquid medium. Quantification of undecylprodigiosin (RED) production allowed us to detect an increase of more than 10-fold of RED in the Δ*abrB* strain from 36 to 68 h (Figure 2A). Quantification of ACT also showed a 20-fold increment in the mutant strain compared to the wild-type at 60 h and a 1.5-fold increase at 68 h (Figure 2A). No differences were detected between both strains with respect to growth rate nor to the germination time (8 h) under this culture condition (Figure 2B).

These results highlight the role of the TCS AbrB1/B2 as a negative regulator of antibiotic production under these conditions with no apparent effect on growth or morphological differentiation.

### Global Transcriptomic Assay: Mutant Δ*abrB* Strain Versus the Wild-Type

To examine the regulation cascade triggered by the AbrB1/B2 system more closely, a global comparative transcriptomic assay between the mutant strain and the wild-type was performed.

The expression pattern of both genes (*abrB1* and *abrB2*) was determined by RT-qPCR in the wild-type M145 strain at 24, 36, 48, and 60 h in NMMP liquid medium. Both genes were expressed over the time course studied, although an expression peak was observed at the earlier time of 24 h (mid exponential growth phase) which decreased at 36 h (early stationary growth phase) until reaching a relative constant expression at 48 and 60 h (Supplementary Figure 5). Consequently, an RNA-Seq analysis was performed in NMMP medium at 24 and 36 h, the time points where expression of the *abrB1/B2* system was the highest.

**FIGURE 3:**
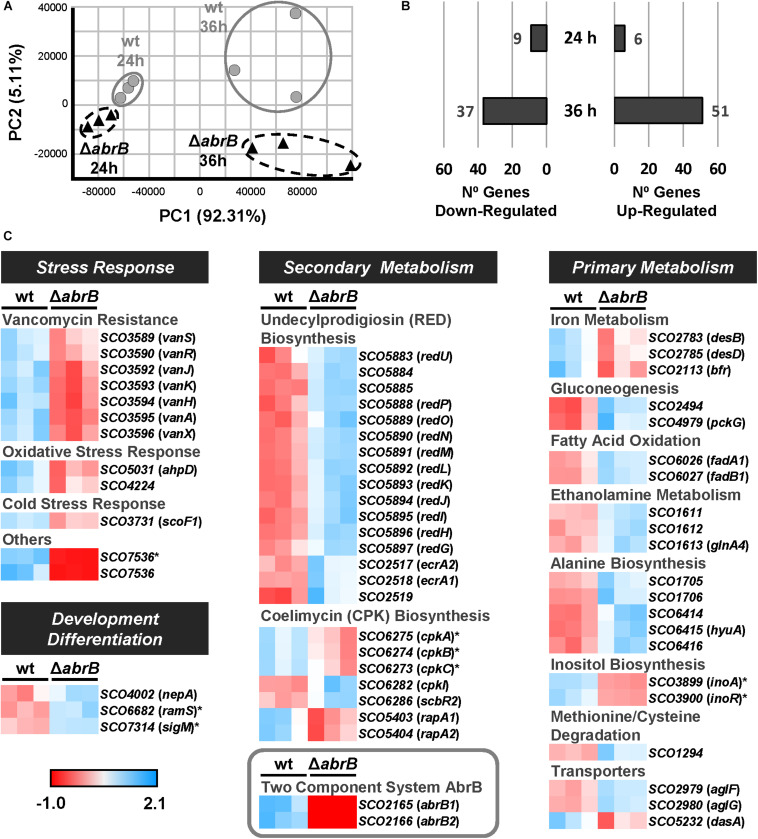
Overview of RNA-Seq assay. **(A)** Principal component analysis (PCA) of the RNA-Seq samples: triplicates of the wt *S. coelicolor* M145 (wt; gray circles) and the mutant Δ*abrB* (black triangles) at 24 and 36 h. **(B)** Number of up-regulated (FC > 2) and down-regulated (FC < –2) genes in the mutant strain relative to the wt at 24 and 36 h. The selected genes passed the filter of padj ≤ 0.05. **(C)** Heatmaps of standardized TPMs (transcripts per million) from the differentially expressed genes between *S. coelicolor* M145 wt and mutant Δ*abrB* strains at 24 h (marked with asterisks) and 36 h. The genes with unknown functions or biological roles have not been included.

The samples clearly showed a differential expression clustering for each strain at each of the time points using principal component analysis (PCA) (Figure 3A). To perform the global analysis, the normalized data were filtered according to the following criteria: padj ≤ 0.05 and fold change of two (log_2_FC ≤ -1; log_2_FC ≥ 1). At 24 h, 15 genes were differentially expressed between both strains: 6 up-regulated and 9 down-regulated in the mutant Δ*abrB* relative to the wild-type (Figure 3B and Supplementary Table 4). At 36 h, 88 genes were differentially expressed between both strains: 51 up-regulated and 37 down-regulated in the mutant Δ*abrB* relative to the wild-type (Figure 3B and Supplementary Table 5).

The differentially expressed genes belonged to several biological processes: stress response, primary and secondary metabolism, and development/differentiation (Figure 3C).

#### Stress Response

The mutant Δ*abrB* strain exhibited a down-regulation of several response systems to different types of stress: antibiotic resistance, oxidative stress and cold shock.

##### Vancomycin resistance

The complete gene cluster of vancomycin resistance, *vanSRJKHAX* (*SCO3589/90/92/93/94/95/96*), was downregulated in the mutant at 36 h. This cluster is divided into four transcriptional units: *vanSR* (TCS which regulates cluster expression in response to the presence of vancomycin), *vanJ*, *vanK*, and *vanHAX* (enzymes that lead to vancomycin resistance through the modification of the D-Ala-D-Ala terminus of Lipid II, a cell wall precursor, which makes this compound unrecognizable to the antibiotic) (Hong et al., 2004; Hutchings et al., 2006; Koteva et al., 2010).

##### Oxidative stress response

In the mutant strain various genes related to stress response were down-regulated at 36 h: *ahpD* (*SCO5031*) and *SCO4224*. AhpD belongs to the alkyl-hydroperoxide-reductase system, one of the most relevant antioxidant systems of *S. coelicolor* (Hahn et al., 2002). In the case of *SCO4224*, although its product has not yet been characterized, its deletion restricted the growth of the closely specie *S. lividans* in the presence of oxidative stress (Darbon et al., 2012).

##### Others

The gene *scoF1* (*SCO3731*), which encodes a potential cold-shock protein quite similar to CspA (Kormanec and Sevcikova, 2000), was down-regulated at 36 h in the Δ*abrB* strain.

Finally, the differential expression exhibited by *SCO7536* was particularly noteworthy, with the highest fold-changes at both 24 h (FC -17) and 36 h (FC -27). This gene seems to encode a potential drug exporter, although at present there are no studies regarding this gene.

#### Secondary Metabolism

##### Undecylprodigiosin (RED) biosynthesis

According to the phenotypic analysis previously described, most of the genes of the *red* gene cluster, which manage RED production (Stankovic et al., 2014) were up-regulated at 36 h in the mutant strain. These genes were *redUPONMLKJIHG* (*SCO5883/88/89/90/91/92/93/94/95/96/97*) and *SCO5884/85*, whose role in the biosynthetic pathway are unknown. The cluster-specific regulator *redD* (*SCO5877*) did not pass the filters threshold of differential expression. However, the operon *SCO2517-19* which contains *ecrA1/A2* (*SCO2518/17*), a TCS that positively regulates RED biosynthesis (Li et al., 2004), was up-regulated in the mutant strain at 36 h.

##### Coelimycin (CPK) biosynthesis

CPK production was also affected in the Δ*abrB* mutant strain, where several related genes were differentially expressed: *cpkABC* (*SCO6275/74/73*), *cpkI* (*SCO6282*), *scbR2* (*SCO6286*) and *rapA1/A2* (*SCO5403/04*). The core genes of the CPK biosynthetic gene cluster, *cpkABC*, were down-regulated in the mutant strain at 24 h, while *cpkI*, involved in post-polyketide tailoring (Pawlik et al., 2007; Bednarz et al., 2019), was up-regulated in the mutant strain at 36 h. The regulator *scbR2* was also up-regulated in the mutant strain at 36 h. The main role of this gene is to coordinate the production of coelimycin, undecylprodigiosin, actinorhodin and calcium-dependent antibiotics, acting as a repressor of coelimycin biosynthesis and an activator of the remaining compounds (Xu et al., 2010; Bednarz et al., 2019), which was in accordance with the results obtained in this work. The TCS *rapA1/A2* was down-regulated in the mutant strain at 36 h; this system acts as a positive regulator of coelimycin and actinorhodin production (Lu et al., 2007).

##### Actinorhodin (ACT) biosynthesis

Although increased ACT production was phenotypically observed in the mutant, few genes related to this process passed the filters established, most probably due to the early time points at which RNA-Seq was performed. However, the previously mentioned regulator ScbR2 and the TCS RapA are known to be related to ACT production. ScbR2 acts as a positive regulator of ACT biosynthesis, and its over-expression in the mutant strain agreed with this phenotype. On the contrary, RapA (also a positive regulator of this process) was under-expressed in the mutant, which could mean ScbR2 has a dominant effect over RapA. Aside from these regulatory systems, there were several genes that did not pass through the filters (0.05 < padj < 0.08) but were still relevant to the analysis: *actI-ORF3* (*SCO5089*), *actVII* (*SCO5090*), *actIV* (*SCO5091*), and *actVB* (*SCO5092*) (Supplementary Table 6). These genes (up-regulated in the mutant strain) belong to the *act* gene cluster and encode enzymes related to ACT production (Okamoto et al., 2009).

#### Primary Metabolism

##### Iron metabolism

Systems for iron uptake (*desBD*) and iron storage (*bfr*) were down-regulated at 36 h in the mutant strain. The genes *desB* (*SCO2783*) and *desD* (*SCO2785*) participate in deferoxamine biosynthesis, siderophores that mediate iron uptake (Barona-Gómez et al., 2004). The *bfr* gene encodes a bacterioferritin, an intracellular iron-storage protein.

##### Gluconeogenesis

The mutant Δ*abrB* exhibited an over-expression of genes *SCO2494* and *pckG* (*SCO4979*), implicated in the early steps of gluconeogenesis at 36 h. Both genes form part of the phosphoenolpyruvate-pyruvate-oxaloacetate node (PEP-PYR-OXA), a central point in carbon metabolism. The gene *SCO2494* encodes a pyruvate phosphate dikinase, while the gene *pckG* encodes a phosphoenolpyruvate carboxykinase (Llamas-Ramírez et al., 2020).

##### Fatty acid oxidation

The *fadA1/B1* (*SCO6026/27*) system was up-regulated at 36 h in the mutant strain. This system is involved in the final steps of fatty acid β-oxidation (Menéndez-Bravo et al., 2017).

##### Ethanolamine metabolism

The operon *SCO1611-13* was up-regulated in the mutant strain at 36 h. Although the entire operon is related with ethanolamine metabolism, only *glnA4* (*SCO1613*) has been studied more in depth. GlnA4 is a γ-glutamyl-ethanolamine synthetase, an enzyme that allows *S. coelicolor* to use ethanolamine as an alternative nitrogen/carbon source (Rexer et al., 2006; Krysenko et al., 2019).

##### Alanine biosynthesis

The mutant Δ*abrB* showed an over-expression at 36 h of genes implicated in the alanine biosynthesis: SCO1705/06 and SCO6414/15/16, according to several databases (KEGG^4^, BioCyc^5^, etc.).

##### Inositol biosynthesis

The operon *inoAR* (*SCO3899/3900*) was down-regulated at 24 h in the mutant Δ*abrB*. This operon which leads inositol biosynthesis is formed by the regulator InoR and the myo-inositol phosphate synthase InoA. Although inositol is usually related to the process of differentiation as a phospholipid precursor (Zhang et al., 2010, 2012), this molecule also has a role as a mycothiol precursor, a compound that protects *S. coelicolor* from oxidative stress, like glutathione in other organisms (Park and Roe, 2008).

##### Methionine/cysteine degradation

The gen *SCO1294* was up-regulated in *S. coelicolor* at 36 h. Even though this gene has not been studied in *S. coelicolor*, its homolog in *S. avermitilis* (*SAV7062*) has been described as a L-methionine-γ-liase that degrades methionine, cysteine, and derivatives (Kudou et al., 2015).

##### Transporters

Several transporter systems were differentially expressed between the wild-type and the mutant strains. The operon *aglFG* (*SCO2979/2980*) was up-regulated in the mutant at 36 h, and *in silico* studies suggest the operon constitutes an α-glucosides permease (Bertram et al., 2004). The gene *dasA* (*SCO5232*) was down-regulated in the mutant strain at 36 h, where DasA takes part in the uptake system of N,N′-diacetyl-chitobiose and similar molecules such as N-acetyl-glucosamine (Saito et al., 2007).

#### Development/Differentiation

The genes *nepA* (*SCO4002*), *ramS* (*SCO6682*), and *sigM* (*SCO7314*) were also over-expressed in the mutant strain. NepA is a structural protein of the cell wall and is related to spore dormancy (Dalton et al., 2007; de Jong et al., 2009). RamS is the SapB precursor, a morphogenetic peptide in aerial mycelium development (Kodani et al., 2004). SigM, or Sigma Factor M, is the last step in the regulation cascade of Sigma Factor B and participates in differentiation and in the protection against oxidative and osmotic stress (Lee et al., 2005).

In order to validate the RNA-Seq data by RT-qPCR, three up-regulated genes, *ecrA1* (*SCO2518*), *redG* (*SCO5897*), and *mfnB* (*SCO6440*) and three down-regulated genes, *bfr* (*SCO2113*), *vanJ* (*SCO3592*), and *SCO7536*, were selected. The fold-changes obtained were similar to those obtained by RNA-Seq for the down-regulated genes, but there were more differences in the fold change obtained by RT-qPCR than by RNA-Seq (Supplementary Table 7) for the up-regulated genes.

Finally, no potential motifs were found implied in the regulation of this set of genes by AbrB1/B2, which could mean that the DNA recognition motif of the RR AbrB1 was highly degenerated.

### AbrB1/B2 Contributes to Vancomycin Resistance

Although AbrB1/B2 seemed to control the basal levels of the vancomycin resistance gene cluster (*vanSRJKHAX*), as can be seen in the RNA-Seq results, this did not necessarily be affecting the resistance potential of *S. coelicolor* in the presence of vancomycin, because this resistance operon (*van*) mainly responds to the presence of the antibiotic through the VanRS TCS.

**FIGURE 4:**
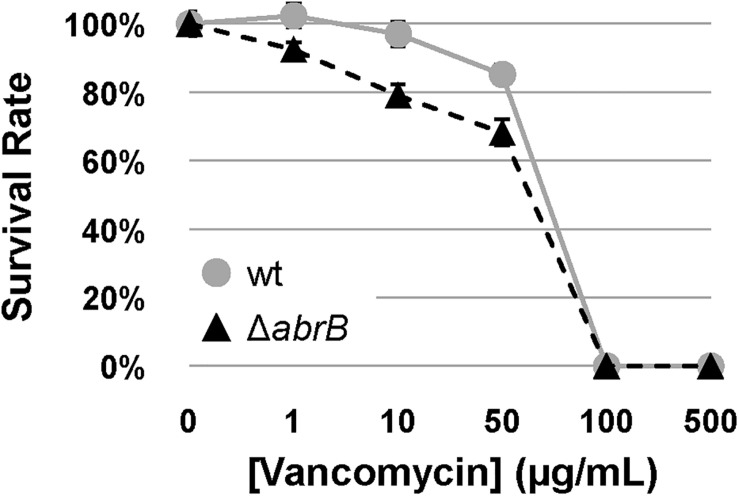
Vancomycin survival assay. Survival assay of spores from *S. coelicolor* M145 wt (wt; gray circles) and mutant Δ*abrB* strains (black triangles) against different concentrations of vancomycin in NMMP plates. Error bars show the standard deviation of quadruplicate assays.

Therefore, to determine the role of AbrB1/B2 in this process, the survival rates of specific spore concentrations of the M145 wild-type strain and the Δ*abrB* mutant were examined on NMMP solid medium with increasing concentrations of vancomycin. The deletion of the AbrB1/B2 system triggered a higher susceptibility of *S. coelicolor* to vancomycin (Figure 4). This result confirmed, according to the alteration of the basal levels of the vancomycin resistance gene cluster (*van*), that AbrB1/B2 also contributes to vancomycin resistance in *S. coelicolor*.

### AbrB1/B2 Is a Homolog of VraR/S and LiaR/S

The role of AbrB1/B2 in vancomycin resistance led us to look for their homologs in clinically relevant bacteria, such as enterococci, whose vancomycin-resistance strains (VREs) constitute a serious threat to public health. The search and analysis were made based on the RRs, which are much better characterized than HKs.

In the literature, the only system described to regulate VanR/S (like AbrB1/B2 in *S. coelicolor*, as observed in this study) was VraR/S in *S. aureus* (Qureshi et al., 2014). The pairwise alignment of the amino acid sequences of AbrB1 and VraR showed an identity and similarity over 30 and 50%, respectively, similar to that described for other homologs found in both species such as VanR (Supplementary Table 8). Furthermore, a maximum likelihood analysis of AbrB1 (from *S. coelicolor*) and VraR (from *S. aureus*), plus other RRs involved in vancomycin resistance in *S. aureus*, like VanR, WalR, and GraR, revealed that VraR had a closer relationship with AbrB1 than with the other staphylococci RRs (Supplementary Figure 2). Similar results were obtained when AbrB1 was compared with LiaR from *E. faecium* and *E. faecalis*, a closely related homolog of VraR (despite the sequence differences, both VraR and LiaR share exactly the same tertiary structure, experimentally obtained, and fulfill the same biological function).

**FIGURE 5:**
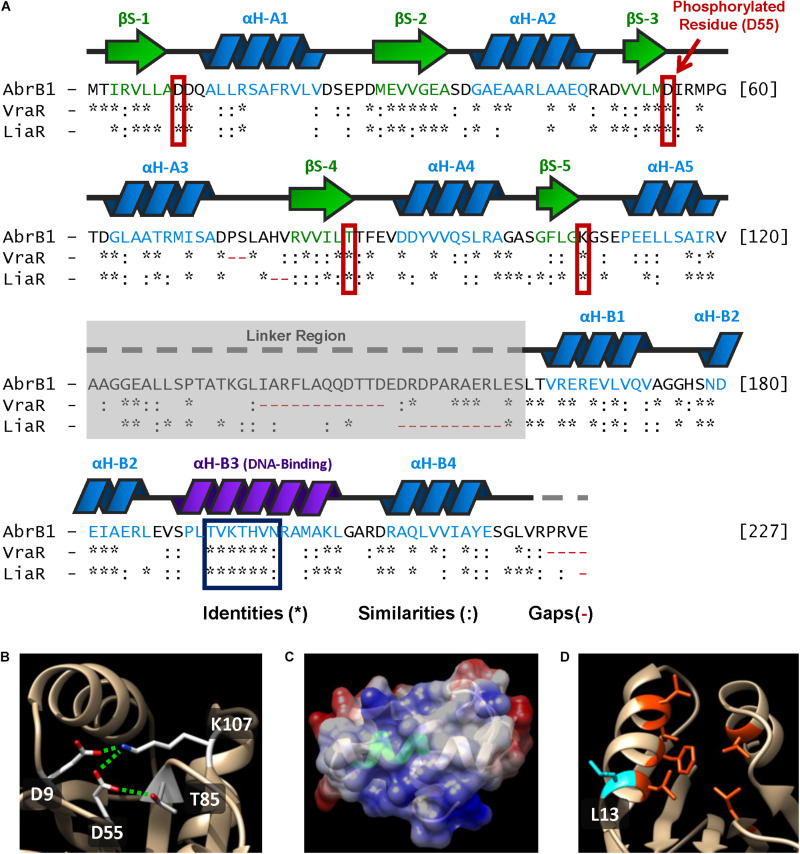
Structural features of AbrB1 and its comparison with VraR and LiaR. **(A)** Sequence of the RR AbrB1. Secondary structures are shown above the amino acid sequence (beta-strands in green, and alpha-helices in blue). Comparison with VraR from *S. aureus* and LiaR from *E. faecium* is shown below the AbrB1 sequence; identities (*), similarities (:), and gaps (-) are indicated (pairwise alignment was performed in *EMBOSS Needle* applying a BLOSUM62 substitution matrix). Active site residues are squared in red, while conserved DNA-recognition site is squared in blue. **(B)** Phosphorylation site in AbrB1 structural model: D55 (phosphorylated residue), D9, K107, and T85. Interactions between residues (inactive form) are shown as dashed green lines. **(C)** DNA-binding helix in AbrB1 structural model. Conserved recognition site is shown in green. Electrostatic surface is represented (positive charge as blue, and negative charge as red). **(D)** Hydrophobic “pocket” in REC domain for the dimerization process. The residues that conform this “pocket” are shown in orange (Leu14, Phe18, Leu 21, Val22, Leu84, and Leu114); protuberant L13 (which interacts with the “pocket” of the other protomer) is shown in blue.

Relevant structural data have been published on VraR and LiaR (Donaldson, 2008; Leonard et al., 2013; Davlieva et al., 2015, 2016). All the relevant residues identified in both regulators were conserved in AbrB1, both at the amino acid sequence level and at the level of the three-dimensional structure [homology modeling of AbrB1 was performed in *SWISS-MODEL* using the structure of VraR (*4GVP.1.A*) as a template] (Figure 5):

1. Phosphorylation site residues: Asp55 (putative phosphorylated residue), Asp9, Thr85, and Lys107 (residues with which Asp55 interacts in the inactive form, non-phosphorylated) (Figure 5B).
2. DNA recognition site: from Thr192 to Asn198 (Figure 5C). Additionally, the residues that stabilize DNA binding were also conserved in AbrB1: Arg165, Asn179, and Arg209.
3. Hydrophobic “pocket” in REC domain for the dimerization process (Figure 5D). Although in this feature the residues were not completely conserved, the hydrophobicity of this region was maintained: Leu14, Phe18, Leu 21, Val22, Leu84, and Leu114. The protuberant methionine that interacts with the hydrophobic “pocket” of the other protomer in VraR was substituted by leucine (Leu13) in AbrB1, which shows an almost identical hydrophobicity (Finney et al., 1980; Guy, 1985).
4. Hydrophobic nucleus of the alpha-helix α4 in the HTH-LuxR domain for the dimerization process: from Leu212 to Ala216. Like the previous feature, hydrophobicity was conserved although the residues were not identical.
5. The core of REC domain (beta-strands) and the core of HTH-LuxR domain (alpha-helices) show a high degree of conservation.

## Discussion

**FIGURE 6:**
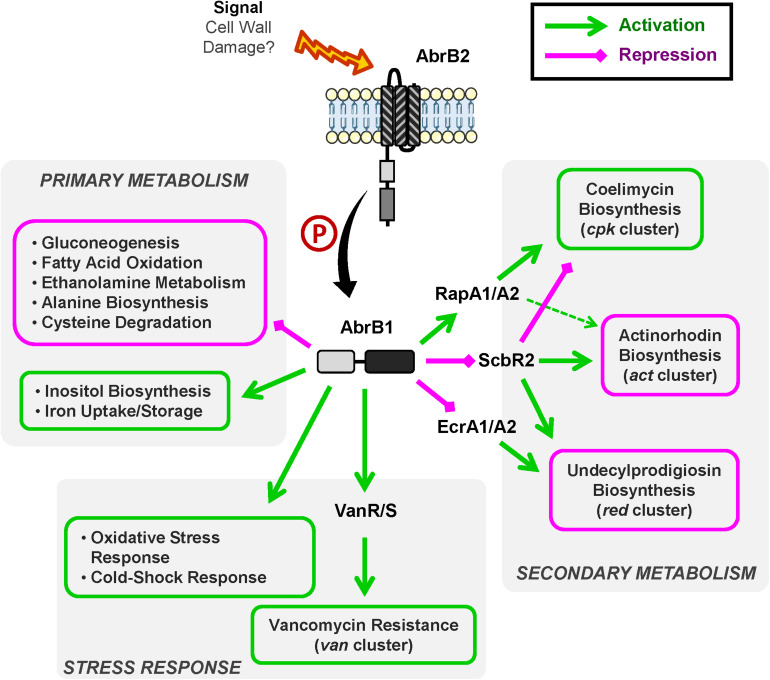
Overview of AbrB1/B2 regulatory cascade. Summary of positively regulated processes (green) and negatively regulated processes (magenta) by the TCS AbrB1/B2.

A summary of AbrB1/B2 regulatory cascade is shown in Figure 6.

### Regulation of Antibiotic Production

The role of AbrB1/B2 as negative regulator of antibiotic biosynthesis has been shown at the phenotypic and transcriptional levels. This TCS is involved in RED and ACT production in *S. coelicolor*. The regulatory cascade controlling these pathways involves the TCS EcrA1/A2 in the case of RED and the regulator ScbR2 in the case of the regulation of both RED and ACT synthesis.

In sum, AbrB1/B2 could be a repressor of EcrA1/A2, a positive regulator of RED biosynthesis (Li et al., 2004), which is linked to the negative regulation of RED production. This regulation could be in part due to cross-talk events, due to the high similarity present between EcrA1/A2 and AbrB1/B2 (the RRs have an identity/similarity in their amino acid sequences of 53 and 66%, respectively). ScbR2 could also be negatively regulated by AbrB1/B2, which would equate to a repression of ACT and RED production and an activation of CPK, because ScbR2 coordinates antibiotic production in *S. coelicolor* as a positive regulator of ACT and RED and a negative regulator of CPK (Xu et al., 2010; Bednarz et al., 2019). Furthermore, the action of ScbR2 seems to be dominant over the TCS RapA1/A2 in the control of ACT production, since RapA1/A2 is a positive regulator of ACT and CPK synthesis (Lu et al., 2007) but is positively regulated by AbrB1/B2.

The negative regulation of antibiotic production by AbrB1/B2 may seem contradictory, as CPK is down-regulated in the absence of this TCS (a regulatory cascade that seems to imply ScbR2 and RapA1 regulators). However, although the classical role of CPK has been attributed to antibiotic activity, this role is unclear from a physiological point of view. Despite the antibiotic properties of coelimycin A, this molecule is highly unstable and spontaneously reacts with compounds present in the medium, such as N-acetylcysteine and glutamate, generating coelimycin P1 and P2 (compounds without antibiotic properties), respectively (Gottelt et al., 2010; Gómez-Escribano et al., 2012).

The down-regulation of antibiotic production by AbrB1/B2 could be, at the physiological level, a strategy to maintain cellular energetics, since both antibiotic synthesis and cell wall remodeling in the vancomycin resistance are processes of high energetic cost.

### Regulation of Vancomycin Resistance

Vancomycin is a glycopeptide antibiotic that inhibits cell wall formation by joining peptidoglycan precursors (Lipid II) and blocking crosslink formation. Vancomycin only affects Gram+ bacteria (this antibiotic is not able to cross the external membrane present in Gram− bacteria), and its clinical importance is key since it is the unique effective treatment against *S. aureus* MRSA. The vancomycin resistance gene cluster (*van*) is present in multiple human pathogens (*S. aureus, E. faecalis*, and *E. faecium*) and actinomycetes (glycopeptide antibiotic producers such as *Amycolatopsis orientalis* and *Streptomyces toyocaensis* and non-producers such as *S. coelicolor*). However, despite the central role of the *van* cluster, other systems like VraR/S and GraR/S also contribute to vancomycin resistance in different ways (Hong et al., 2008).

In this work, the role of AbrB1/B2 as a positive regulator of vancomycin resistance has been shown at the phenotypic and transcriptional levels. This TCS acts as a positive regulator of the *van* cluster and as a result increases vancomycin resistance. The regulation of this resistance mechanism could explain a great part of RNA-Seq results, as will be detailed below:

1. Antibiotic responses, oxidative stress and iron metabolism can be integrated as follows. The presence of antibiotics increases ROS (reactive-oxygen species) levels, which contribute to antibiotic toxicity to bacteria, although the molecular mechanism for this is still under debate. One of the proposed theories suggests that antibiotic action in essential cellular processes (like cell wall synthesis in the case of vancomycin) could generate some dysfunctions in the respiratory chain. This would provoke an increase in the concentration of ROS which would destabilize Fe-S clusters, releasing iron and giving rise to Fenton’s reaction. This would enhance, as positive feedback, the toxic effect of the ROS, and, subsequently, the antibiotic (Ezraty and Barras, 2016). All of this could explain the differential expression of the genes related to oxidative stress (both directly like *ahpD* and indirectly like *inoAR*) and the iron uptake/storage detected in the RNA-Seq analysis.
2. The link between antibiotic resistance and thermal shock is not new. It has been described that ribosomes affecting antibiotics induce heat-shock proteins and cold-shock proteins depending on its mechanism of action (VanBogelen and Neidhardt, 1990). Furthermore, it has been proposed a report system that precisely use thermal-shock response promoters (like the *cspA* promoter) to discover new antibiotics (Bianchi and Baneyx, 1999).
3. Changes in primary metabolism (gluconeogenesis, fatty acids oxidation, ethanolamine metabolism, etc.) could be also attributed to vancomycin resistance and cell wall remodeling. Alterations in carbon and fatty acids metabolism has been previously reported in a transcriptomic assay related to VraR (homolog of AbrB1, as it was shown in this study) in vancomycin-resistance *S. aureus* strains (Kuroda et al., 2000). For example, vancomycin-resistance is based on Lipid II modification (D-Ala-D-Ala terminus is substituted by D-Ala-D-Lac), therefore, the overexpression of genes related to alanine biosynthesis in the mutant Δ*abrB* is probably a consequence of the change in alanine requirements.
4. Despite the absence of significant changes in development and differentiation (wild-type and mutant strain show a similar growth, germination time, etc.), *nepA*, *ramS*, and *sigM* were differentially expressed in the RNA-Seq assay. Changes in the expression of *nepA* could be a side effect of cell wall remodeling due to vancomycin resistance, since this gene encodes a cell wall structural protein. In the case of *ramS*, the absence of phenotypic changes in minimal medium has been previously described, since only chaplins are needed for aerial mycelium formation in this medium (Kodani et al., 2004; Capstick et al., 2007) and its differential expression could also be provoked by cell-wall remodeling. Finally, *sigM*, a sigma factor related to differentiation and protection against osmotic and oxidative stress (Lee et al., 2005; Gaskell et al., 2007), could only be manifesting its role in oxidative stress in these conditions, coordinating the alternative action of alkyl-hydroperoxide-reductase and catalase systems.

The homology of AbrB1/B2 to VraR/S and LiaR/S also supports its role in the mechanism of vancomycin resistance. Both VraR/S (from *S. aureus*) and LiaR/S (present in a multitude of bacteria such as *E. faecium*, *Bacillus subtilis*, *Streptococcus mutans*, etc.) are involved in cell envelope stress responses. These systems are not only induced by vancomycin, but also other cell wall active antibiotics (like β-lactams in the case of VraR/S) and other perturbations in cell wall integrity. This suggest that these TCSs detect cell wall damage rather than the antibiotics themselves. Thus, they have been cataloged as “sentinel” systems capable of sensing cell wall perturbations, although the specific activation signal is not clear. Both VraR/S and LiaR/S respond to cell envelope stress through the activation of systems involved in the maintenance of cell wall integrity (such as the peptidoglycan biosynthetic pathway). Mutations and overexpression of these systems have been related to VREs (for example, VISA strains of *S. aureus* show high expression levels of VraR/S that provoke cell wall thickening) (Kuroda et al., 2003; Mascher et al., 2004; Gardete et al., 2006; Yin et al., 2006; Belcheva and Golemi-Kotra, 2008; Suntharalingam et al., 2009; Klinzing et al., 2013; Miller et al., 2014; Qureshi et al., 2014; Draper et al., 2015; Chen et al., 2016; Tierney and Rather, 2019).

The connection between cell wall integrity and vancomycin resistance has been previously evidenced in *S. coelicolor*. In this organism the resistance to vancomycin is phosphate-dependent: high concentrations of inorganic phosphate in the media increase the susceptibility to this antibiotic. Surprisingly, this process is not mediated by the main regulator of phosphate metabolism, the TCS PhoRP (Santos-Beneit and Martín, 2013), but it seems to be related with the cell wall metabolism, since the deletion or mutation of genes implied in this process (*SCO2594* and *SCO1213*; this last one is a *gatD* homolog) overcomes the phosphate effect on vancomycin resistance (Santos-Beneit et al., 2014; Santos-Beneit, 2018).

Our working hypothesis for future work is that the role of AbrB1/B2 could be to detect cell wall damage (similar to VraR/S and LiaR/S) and regulate systems that contribute to the maintain of cell wall integrity such as the *van* cluster whose TCS (VanR/S) directly detects vancomycin molecules (Koteva et al., 2010; Novotna et al., 2015; Lockey et al., 2020) and promotes cell wall remodeling. Thus, a preliminary experiment testing the susceptibility to penicillin G agrees with the working hypothesis, being the Δ*abrB* mutant more sensitive to this ß-lactam (Supplementary Figure 6).

### Biotechnological Applications

The results obtained in this work are interesting from a biotechnological perspective since, on one hand, this TCS is a negative regulator of antibiotic production, and its high degree of conservation throughout *Streptomyces* spp. makes it a valuable tool for improving antibiotic production and the discovery of cryptic metabolites with antibiotic action. On the other hand, AbrB1/B2 contributes to vancomycin resistance and is a homolog of VraR/S and LiaR/S, important regulators in clinically relevant antibiotic-resistant bacteria (*S. aureus* MRSA and vancomycin-resistance enterococci, VRE, are considered serious threats to public health that require prompt and sustained action according to the CDC in its latest report on antibiotic resistance). Therefore, the study of AbrB1/B2 could provide new insight into the mechanism of this type of resistance.

## Data Availability Statement

The datasets presented in this study can be found in online repositories. The names of the repository/repositories and accession number(s) can be found below: https://www.ncbi.nlm.nih.gov/, PRJNA637401.

## Author Contributions

RSN and SA conducted the experiments. RSN, JA, and MD analyzed the results. RIS, MD, SA, and RSN designed the experiments and wrote the manuscript. All authors have read and approved the final manuscript.

## Conflict of Interest

The authors declare that the research was conducted in the absence of any commercial or financial relationships that could be construed as a potential conflict of interest.

## Footnotes

1 https://string-db.org

2 http://smart.embl-heidelberg.de/

3 http://www.p2cs.org/

4 https://www.genome.jp/kegg/

5 https://biocyc.org/

## References

[B1] Álvarez A. F. Barba-Ostria C. Silva-Jiménez H. Georgellis D. (2016). Organization and mode of action of two component system signaling circuits from the various kingdoms of life. *Environ. Microbiol.* 18 3210–3226. 10.1111/1462-2920.13397 27235890

[B2] Antoraz S. Rico S. Rodríguez H. Sevillano L. Alzate J. F. Santamaría R. I. (2017). The orphan response regulator Aor1 is a new relevant piece in the complex puzzle of *Streptomyces coelicolor* antibiotic regulatory network. *Front. Microbiol.* 8:2444. 10.3389/fmicb.2017.02444 29312165 PMC5733086

[B3] Barakat M. Ortet P. Whitworth D. E. (2011). P2CS: a database of prokaryotic two-component systems. *Nucleic Acids Res.* 39 D771–D776. 10.1093/nar/gkq1023 21051349 PMC3013651

[B4] Baral B. Akhgari A. Metsa-Ketela M. (2018). Activation of microbial secondary metabolic pathways: avenues and challenges. *Synth. Syst. Biotechnol.* 3 163–178. 10.1016/j.synbio.2018.09.001 30345402 PMC6190515

[B5] Barka E. A. Vatsa P. Sanchez L. Gaveau-Vaillant N. Jacquard C. Klenk H. P. (2016). Taxonomy, physiology, and natural products of actinobacteria. *Microbiol. Mol. Biol. Rev.* 80 1–43. 10.1128/mmbr.00019-15 26609051 PMC4711186

[B6] Barona-Gómez F. Wong U. Giannakopulos A. E. Derrick P. J. Challis G. L. (2004). Identification of a cluster of genes that directs desferrioxamine biosynthesis in *Streptomyces coelicolor* M145. *J. Am. Chem. Soc.* 126 16282–16283. 10.1021/ja045774k 15600304

[B7] Becerril A. Álvarez S. Braña A. F. Rico S. Díaz M. Santamaría R. I. (2018). Uncovering production of specialized metabolites by *Streptomyces argillaceus*: activation of cryptic biosynthesis gene clusters using nutritional and genetic approaches. *PLoS One* 13:e0198145. 10.1371/journal.pone.0198145 29795673 PMC5993118

[B8] Bednarz B. Kotowska M. Pawlik K. J. (2019). Multi-level regulation of coelimycin synthesis in *Streptomyces coelicolor* A3(2). *Appl. Microbiol. Biotechnol.* 103 6423–6434. 10.1007/s00253-019-09975-w 31250060 PMC6667686

[B9] Belcheva A. Golemi-Kotra D. (2008). A close-up view of the VraSR two-component system. A mediator of *Staphylococcus aureus* response to cell wall damage. *J. Biol. Chem.* 283 12354–12364. 10.1074/jbc.M710010200 18326495

[B10] Bertram R. Schlicht M. Mahr K. Nothaft H. Saier M. H. Jr. Titgemeyer F. (2004). In silico and transcriptional analysis of carbohydrate uptake systems of *Streptomyces coelicolor* A3(2). *J. Bacteriol.* 186 1362–1373. 10.1128/jb.186.5.1362-1373.2004 14973030 PMC344420

[B11] Bhagirath A. Y. Li Y. Patidar R. Yerex K. Ma X. Kumar A. (2019). Two component regulatory systems and antibiotic resistance in gram-negative pathogens. *Int. J. Mol. Sci.* 20:1781. 10.3390/ijms20071781 30974906 PMC6480566

[B12] Bianchi A. A. Baneyx F. (1999). Stress responses as a tool to detect and characterize the mode of action of antibacterial agents. *Appl. Environ. Microbiol.* 65 5023–5027. 10.1128/aem.65.11.5023-5027.1999 10543818 PMC91676

[B13] Blaskovich M. A. Butler M. S. Cooper M. A. (2017). Polishing the tarnished silver bullet: the quest for new antibiotics. *Essays Biochem.* 61 103–114. 10.1042/EBC20160077 28258234 PMC5869247

[B14] Caille O. Rossier C. Perron K. (2007). A copper-activated two-component system interacts with zinc and imipenem resistance in *Pseudomonas aeruginosa*. *J. Bacteriol.* 189 4561–4568. 10.1128/jb.00095-07 17449606 PMC1913472

[B15] Capstick D. S. Willey J. M. Buttner M. J. Elliot M. A. (2007). SapB and the chaplins: connections between morphogenetic proteins in *Streptomyces coelicolor*. *Mol. Microbiol.* 64 602–613. 10.1111/j.1365-2958.2007.05674.x 17462011

[B16] CDC (2019). *CDC. Antibiotic Resistance Threats in the United States.* Atlanta, GA: U.S. Department of Health and Human Services.

[B17] Chang H. M. Chen M. Y. Shieh Y. T. Bibb M. J. Chen C. W. (1996). The cutRS signal transduction system of *Streptomyces lividans* represses the biosynthesis of the polyketide antibiotic actinorhodin. *Mol. Microbiol.* 21 1075–1085. 8885276

[B18] Chen H. Xiong Z. Liu K. Li S. Wang R. Wang X. (2016). Transcriptional profiling of the two-component regulatory system VraSR in *Staphylococcus aureus* with low-level vancomycin resistance. *Int. J. Antimicrob. Agents* 47 362–367. 10.1016/j.ijantimicag.2016.02.003 27084050

[B19] Chen V. B. Arendall W. B. III Headd J. J. Keedy D. A. Immormino R. M. Kapral G. J. (2010). MolProbity: all-atom structure validation for macromolecular crystallography. *Acta Crystallogr. D Biol. Crystallogr.* 66(Pt 1), 12–21. 10.1107/S0907444909042073 20057044 PMC2803126

[B20] Dalton K. A. Thibessard A. Hunter J. I. Kelemen G. H. (2007). A novel compartment, the ‘subapical stem’ of the aerial hyphae, is the location of a sigN-dependent, developmentally distinct transcription in *Streptomyces coelicolor*. *Mol. Microbiol.* 64 719–737. 10.1111/j.1365-2958.2007.05684.x 17462019

[B21] Darbon E. Martel C. Nowacka A. Pegot S. Moreau P. L. Virolle M. J. (2012). Transcriptional and preliminary functional analysis of the six genes located in divergence of *phoR/phoP* in *Streptomyces lividans*. *Appl. Microbiol. Biotechnol.* 95 1553–1566. 10.1007/s00253-012-3995-2 22466952

[B22] Davlieva M. Shi Y. Leonard P. G. Johnson T. A. Zianni M. R. Arias C. A. (2015). A variable DNA recognition site organization establishes the LiaR-mediated cell envelope stress response of enterococci to daptomycin. *Nucleic Acids Res.* 43 4758–4773. 10.1093/nar/gkv321 25897118 PMC4482077

[B23] Davlieva M. Tovar-Yanez A. DeBruler K. Leonard P. G. Zianni M. R. Arias C. A. (2016). An adaptive mutation in *Enterococcus faecium* LiaR associated with antimicrobial peptide resistance mimics phosphorylation and stabilizes LiaR in an activated state. *J. Mol. Biol.* 428 4503–4519. 10.1016/j.jmb.2016.09.016 27670715 PMC5085866

[B24] de Jong W. Manteca A. Sanchez J. Bucca G. Smith C. P. Dijkhuizen L. (2009). NepA is a structural cell wall protein involved in maintenance of spore dormancy in *Streptomyces coelicolor*. *Mol. Microbiol.* 71 1591–1603. 10.1111/j.1365-2958.2009.06633.x 19222756

[B25] Donaldson L. W. (2008). The NMR structure of the *Staphylococcus aureus* response regulator VraR DNA binding domain reveals a dynamic relationship between it and its associated receiver domain. *Biochemistry* 47 3379–3388. 10.1021/bi701844q 18293926

[B26] Draper L. A. Cotter P. D. Hill C. Ross R. P. (2015). Lantibiotic resistance. *Microbiol. Mol. Biol. Rev.* 79 171–191. 25787977 10.1128/MMBR.00051-14 PMC4394878

[B27] Ezraty B. Barras F. (2016). The ‘liaisons dangereuses’ between iron and antibiotics. *FEMS Microbiol. Rev.* 40 418–435. 10.1093/femsre/fuw004 26945776

[B28] Fernández-Martínez L. T. Santos-Beneit F. Martín J. F. (2012). Is PhoR-PhoP partner fidelity strict? PhoR is required for the activation of the *pho* regulon in *Streptomyces coelicolor*. *Mol. Genet. Genomics* 287 565–573. 10.1007/s00438-012-0698-4 22643908

[B29] Finney J. L. Gellatly B. J. Golton I. C. Goodfellow J. (1980). Solvent effects and polar interactions in the structural stability and dynamics of globular proteins. *Biophys. J.* 32 17–33. 10.1016/s0006-3495(80)84913-7 7248447 PMC1327251

[B30] Gardete S. Wu S. W. Gill S. Tomasz A. (2006). Role of VraSR in antibiotic resistance and antibiotic-induced stress response in *Staphylococcus aureus*. *Antimicrob. Agents Chemother.* 50 3424–3434. 10.1128/aac.00356-06 17005825 PMC1610096

[B31] Gaskell A. A. Crack J. C. Kelemen G. H. Hutchings M. I. Le Brun N. E. (2007). RsmA is an anti-sigma factor that modulates its activity through a [2Fe-2S] cluster cofactor. *J. Biol. Chem.* 282 31812–31820. 10.1074/jbc.M705160200 17766240

[B32] GLASS (2020). *Global Antimicrobial Resistance Surveillance System (GLASS) Report: Early Implementation 2020.* Geneva: World Health Organization.

[B33] Gómez-Escribano J. P. Alt S. Bibb M. J. (2016). Next generation sequencing of actinobacteria for the discovery of novel natural products. *Mar. Drugs* 14:78. 10.3390/md14040078 27089350 PMC4849082

[B34] Gómez-Escribano J. P. Song L. Fox D. Yeo V. Bibb M. Challis G. (2012). Structure and biosynthesis of the unusual polyketide alkaloid coelimycin P1, a metabolic product of the *cpk* gene cluster of *Streptomyces coelicolor* M145. *Chem. Sci.* 3 2716–2720. 10.1039/c2sc20410j

[B35] Gottelt M. Kol S. Gómez-Escribano J. P. Bibb M. Takano E. (2010). Deletion of a regulatory gene within the cpk gene cluster reveals novel antibacterial activity in *Streptomyces coelicolor* A3(2). *Microbiology* 156(Pt 8), 2343–2353. 10.1099/mic.0.038281-0 20447997

[B36] Green M. R. Sambrook J. (2012). *Molecular Cloning: a Laboratory Manual*, 4th Edn New York, NY: Cold Spring Harbor Laboratory Press.

[B37] Guy H. R. (1985). Amino acid side-chain partition energies and distribution of residues in soluble proteins. *Biophys. J.* 47 61–70. 10.1016/s0006-3495(85)83877-7 3978191 PMC1435068

[B38] Hahn J. S. Oh S. Y. Roe J. H. (2002). Role of OxyR as a peroxide-sensing positive regulator in *Streptomyces coelicolor* A3(2). *J. Bacteriol.* 184 5214–5222. 10.1128/jb.184.19.5214-5222.2002 12218006 PMC137946

[B39] Hong H. J. Hutchings M. I. Buttner M. J. (2008). Vancomycin resistance VanS/VanR two-component systems. *Adv. Exp. Med. Biol.* 631 200–213. 10.1007/978-0-387-78885-2_14 18792691

[B40] Hong H. J. Hutchings M. I. Neu J. M. Wright G. D. Paget M. S. Buttner M. J. (2004). Characterization of an inducible vancomycin resistance system in *Streptomyces coelicolor* reveals a novel gene (vanK) required for drug resistance. *Mol. Microbiol.* 52 1107–1121. 10.1111/j.1365-2958.2004.04032.x 15130128

[B41] Hutchings M. I. Hong H. J. Buttner M. J. (2006). The vancomycin resistance VanRS two-component signal transduction system of *Streptomyces coelicolor*. *Mol. Microbiol.* 59 923–935. 10.1111/j.1365-2958.2005.04953.x 16420361

[B42] Hwang K. S. Kim H. U. Charusanti P. Palsson B. O. Lee S. Y. (2014). Systems biology and biotechnology of *Streptomyces* species for the production of secondary metabolites. *Biotechnol. Adv.* 32 255–268. 10.1016/j.biotechadv.2013.10.008 24189093

[B43] Kieser T. Hopwood D. A. Bibb J. M. Chater K. F. Buttner M. J. (2000). *Practical Streptomyces Genetics.* Norwich: John Innes Foundation.

[B44] Klinzing D. C. Ishmael N. Hotopp J. C. D. Tettelin H. Shields K. R. Madoff L. C. (2013). The two-component response regulator LiaR regulates cell wall stress responses, pili expression and virulence in group B *Streptococcus*. *Microbiology* 159(Pt 7), 1521–1534. 10.1099/mic.0.064444-0 23704792 PMC3749725

[B45] Kodani S. Hudson M. E. Durrant M. C. Buttner M. J. Nodwell J. R. Willey J. M. (2004). The SapB morphogen is a lantibiotic-like peptide derived from the product of the developmental gene *ramS* in *Streptomyces coelicolor*. *Proc. Natl. Acad. Sci. U.S.A.* 101 11448–11453. 10.1073/pnas.0404220101 15277670 PMC509221

[B46] Kormanec J. Sevcikova B. (2000). Identification and transcriptional analysis of a cold shock-inducible gene, *cspA*, in *Streptomyces coelicolor* A3(2). *Mol. Genet. Genomics* 264 251–256. 10.1007/s004380000298 11085264

[B47] Koteva K. Hong H. J. Wang X. D. Nazi I. Hughes D. Naldrett M. J. (2010). A vancomycin photoprobe identifies the histidine kinase VanSsc as a vancomycin receptor. *Nat. Chem. Biol.* 6 327–329. 10.1038/nchembio.350 20383152

[B48] Krysenko S. Matthews A. Okoniewski N. Kulik A. Girbas M. G. Tsypik O. (2019). Initial metabolic step of a novel ethanolamine utilization pathway and its regulation in *Streptomyces coelicolor* M145. *mBio* 10:e00326-19. 10.1128/mBio.00326-19 PMC6529630 31113893

[B49] Kudou D. Yasuda E. Hirai Y. Tamura T. Inagaki K. (2015). Molecular cloning and characterization of l-methionine gamma-lyase from *Streptomyces avermitilis*. *J. Biosci. Bioeng.* 120 380–383. 10.1016/j.jbiosc.2015.02.019 25817696

[B50] Kumar S. Stecher G. Li M. Knyaz C. Tamura K. (2018). MEGA X: molecular evolutionary genetics analysis across computing platforms. *Mol. Biol. Evol.* 35 1547–1549. 10.1093/molbev/msy096 29722887 PMC5967553

[B51] Kuroda M. Kuroda H. Oshima T. Takeuchi F. Mori H. Hiramatsu K. (2003). Two-component system VraSR positively modulates the regulation of cell-wall biosynthesis pathway in *Staphylococcus aureus*. *Mol. Microbiol.* 49 807–821. 10.1046/j.1365-2958.2003.03599.x 12864861

[B52] Kuroda M. Kuwahara-Arai K. Hiramatsu K. (2000). Identification of the up- and down-regulated genes in vancomycin-resistant *Staphylococcus aureus* strains Mu3 and Mu50 by cDNA differential hybridization method. *Biochem. Biophys. Res. Commun.* 269 485–490. 10.1006/bbrc.2000.2277 10708580

[B53] Le S. Q. Gascuel O. (2008). An improved general amino acid replacement matrix. *Mol. Biol. Evol.* 25 1307–1320. 10.1093/molbev/msn067 18367465

[B54] Lee E. J. Karoonuthaisiri N. Kim H. S. Park J. H. Cha C. J. Kao C. M. (2005). A master regulator sigmaB governs osmotic and oxidative response as well as differentiation via a network of sigma factors in *Streptomyces coelicolor*. *Mol. Microbiol.* 57 1252–1264. 10.1111/j.1365-2958.2005.04761.x 16101999

[B55] Leonard P. G. Golemi-Kotra D. Stock A. M. (2013). Phosphorylation-dependent conformational changes and domain rearrangements in *Staphylococcus aureus* VraR activation. *Proc. Natl. Acad. Sci. U.S.A.* 110 8525–8530. 10.1073/pnas.1302819110 23650349 PMC3666669

[B56] Letunic I. Bork P. (2018). 20 years of the SMART protein domain annotation resource. *Nucleic Acids Res.* 46 D493–D496. 10.1093/nar/gkx922 29040681 PMC5753352

[B57] Lewis R. A. Wahab A. Bucca G. Laing E. E. Moller-Levet C. S. Kierzek A. (2019). Genome-wide analysis of the role of the antibiotic biosynthesis regulator AbsA2 in *Streptomyces coelicolor* A3(2). *PLoS One* 14:e0200673. 10.1371/journal.pone.0200673 30969967 PMC6457490

[B58] Li Y. Q. Chen P. L. Chen S. F. Wu D. Zheng J. (2004). A pair of two-component regulatory genes ecrA1/A2 in *S. coelicolor*. *J. Zhejiang Univ. Sci.* 5 173–179. 10.1631/jzus.2004.0173 14674028

[B59] Liu G. Chater K. F. Chandra G. Niu G. Tan H. (2013). Molecular regulation of antibiotic biosynthesis in *Streptomyces*. *Microbiol. Mol. Biol. Rev.* 77 112–143. 10.1128/mmbr.00054-12 23471619 PMC3591988

[B60] Llamas-Ramírez R. Takahashi-Iniguez T. Flores M. E. (2020). The phosphoenolpyruvate-pyruvate-oxaloacetate node genes and enzymes in *Streptomyces coelicolor* M-145. *Int. Microbiol.* 23 429–439. 10.1007/s10123-019-00116-x 31900743

[B61] Lockey C. Edwards R. J. Roper D. I. Dixon A. M. (2020). The extracellular domain of two-component system sensor kinase VanS from *Streptomyces coelicolor* binds vancomycin at a newly identified binding site. *Sci. Rep.* 10:5727. 10.1038/s41598-020-62557-z 32235931 PMC7109055

[B62] Lu Y. Wang W. Shu D. Zhang W. Chen L. Qin Z. (2007). Characterization of a novel two-component regulatory system involved in the regulation of both actinorhodin and a type I polyketide in *Streptomyces coelicolor*. *Appl. Microbiol. Biotechnol.* 77 625–635. 10.1007/s00253-007-1184-5 17899070

[B63] Madeira F. Park Y. M. Lee J. Buso N. Gur T. Madhusoodanan N. (2019). The EMBL-EBI search and sequence analysis tools APIs in 2019. *Nucleic Acids Res.* 47 W636–W641. 10.1093/nar/gkz268 30976793 PMC6602479

[B64] Mascher T. Zimmer S. L. Smith T. A. Helmann J. D. (2004). Antibiotic-inducible promoter regulated by the cell envelope stress-sensing two-component system LiaRS of *Bacillus subtilis*. *Antimicrob. Agents Chemother.* 48 2888–2896. 10.1128/AAC.48.8.2888-2896.2004 15273097 PMC478541

[B65] Mendes M. V. Tunca S. Antón N. Recio E. Sola-Landa A. Aparicio J. F. (2007). The two-component *phoR-phoP* system of *Streptomyces natalensis*: inactivation or deletion of *phoP* reduces the negative phosphate regulation of pimaricin biosynthesis. *Metab. Eng.* 9 217–227. 10.1016/j.ymben.2006.10.003 17142079

[B66] Menéndez-Bravo S. Paganini J. Avignone-Rossa C. Gramajo H. Arabolaza A. (2017). Identification of FadAB complexes involved in fatty acid beta-oxidation in *Streptomyces coelicolor* and construction of a triacylglycerol overproducing strain. *Front. Microbiol.* 8:1428. 10.3389/fmicb.2017.01428 28824562 PMC5539140

[B67] Miller W. R. Munita J. M. Arias C. A. (2014). Mechanisms of antibiotic resistance in enterococci. *Expert Rev. Anti Infect. Ther.* 12 1221–1236. 10.1586/14787210.2014.956092 25199988 PMC4433168

[B68] Mouncey N. J. Otani H. Udwary D. Yoshikuni Y. (2019). New voyages to explore the natural product galaxy. *J. Ind. Microbiol. Biotechnol.* 46 273–279. 10.1007/s10295-018-02122-w 30610411

[B69] Novotna G. B. Kwun M. J. Hong H. J. (2015). *In vivo* characterization of the activation and interaction of the VanR-VanS two-component regulatory system controlling glycopeptide antibiotic resistance in two related *Streptomyces* species. *Antimicrob. Agents Chemother.* 60 1627–1637. 10.1128/AAC.01367-15 26711760 PMC4775988

[B70] Okamoto S. Taguchi T. Ochi K. Ichinose K. (2009). Biosynthesis of actinorhodin and related antibiotics: discovery of alternative routes for quinone formation encoded in the act gene cluster. *Chem. Biol.* 16 226–236. 10.1016/j.chembiol.2009.01.015 19246012

[B71] Park J. H. Roe J. H. (2008). Mycothiol regulates and is regulated by a thiol-specific antisigma factor RsrA and sigma(R) in *Streptomyces coelicolor*. *Mol. Microbiol.* 68 861–870. 10.1111/j.1365-2958.2008.06191.x 18430082

[B72] Pawlik K. Kotowska M. Chater K. F. Kuczek K. Takano E. (2007). A cryptic type I polyketide synthase (*cpk*) gene cluster in *Streptomyces coelicolor* A3(2). *Arch. Microbiol.* 187 87–99. 10.1007/s00203-006-0176-7 17009021

[B73] Perron K. Caille O. Rossier C. Van Delden C. Dumas J. L. Kohler T. (2004). CzcR-CzcS, a two-component system involved in heavy metal and carbapenem resistance in *Pseudomonas aeruginosa*. *J. Biol. Chem.* 279 8761–8768. 10.1074/jbc.M312080200 14679195

[B74] Prieto C. Barrios D. (2019). RaNA-Seq: interactive RNA-Seq analysis from FASTQ files to functional analysis. *Bioinformatics* 10.1093/bioinformatics/btz854 [Epub ahead of print]. 31730197

[B75] Qureshi N. K. Yin S. Boyle-Vavra S. (2014). The role of the Staphylococcal VraTSR regulatory system on vancomycin resistance and *vanA* operon expression in vancomycin-resistant *Staphylococcus aureus*. *PLoS One* 9:e85873. 10.1371/journal.pone.0085873 24454941 PMC3893269

[B76] Raavi, Mishra S. Singh S. (2017). Prevention of OprD regulated antibiotic resistance in *Pseudomonas aeruginosa* biofilm. *Microb. Pathog.* 112 221–229. 10.1016/j.micpath.2017.08.007 28826769

[B77] Rexer H. U. Schaberle T. Wohlleben W. Engels A. (2006). Investigation of the functional properties and regulation of three glutamine synthetase-like genes in *Streptomyces coelicolor* A3(2). *Arch. Microbiol.* 186 447–458. 10.1007/s00203-006-0159-8 16932908

[B78] Rico S. Santamaría R. I. Yepes A. Rodríguez H. Laing E. Bucca G. (2014a). Deciphering the regulon of *Streptomyces coelicolor* AbrC3, a positive response regulator of antibiotic production. *Appl. Environ. Microbiol.* 80 2417–2428. 10.1128/aem.03378-13 24509929 PMC3993177

[B79] Rico S. Yepes A. Rodríguez H. Santamaría J. Antoraz S. Krause E. M. (2014b). Regulation of the AbrA1/A2 two-component system in *Streptomyces coelicolor* and the potential of its deletion strain as a heterologous host for antibiotic production. *PLoS One* 9:e109844. 10.1371/journal.pone.0109844 25303210 PMC4193843

[B80] Rodríguez H. Rico S. Díaz M. Santamaría R. I. (2013). Two-component systems in *Streptomyces*: key regulators of antibiotic complex pathways. *Microb Cell Fact.* 12:127. 10.1186/1475-2859-12-127 24354561 PMC3881020

[B81] Rodríguez H. Rico S. Yepes A. Franco-Echevarría E. Antoraz S. Santamaría R. I. (2015). The two kinases, AbrC1 and AbrC2, of the atypical two-component system AbrC are needed to regulate antibiotic production and differentiation in *Streptomyces coelicolor*. *Front. Microbiol.* 6:450. 10.3389/fmicb.2015.00450 26029189 PMC4428217

[B82] Rose M. D. Winston F. Hieter P. (1990). *Methods in Yeast Genetics: a Laboratory Course Manual.* New York, NY: Cold Spring Harbour Laboratory Press.

[B83] Saito A. Shinya T. Miyamoto K. Yokoyama T. Kaku H. Minami E. (2007). The dasABC gene cluster, adjacent to dasR, encodes a novel ABC transporter for the uptake of N,N’-diacetylchitobiose in *Streptomyces coelicolor* A3(2). *Appl. Environ. Microbiol.* 73 3000–3008. 10.1128/aem.02612-06 17351098 PMC1892892

[B84] Santos-Beneit F. (2015). The Pho regulon: a huge regulatory network in bacteria. *Front. Microbiol.* 6:402. 10.3389/fmicb.2015.00402 25983732 PMC4415409

[B85] Santos-Beneit F. (2018). Genome sequencing analysis of *Streptomyces coelicolor* mutants that overcome the phosphate-depending vancomycin lethal effect. *BMC Genomics* 19:457. 10.1186/s12864-018-4838-z 29898657 PMC6001138

[B86] Santos-Beneit F. Fernández-Martínez L. T. Rodríguez-García A. Martín-Martín S. Ordoñez-Robles M. Yagüe P. (2014). Transcriptional response to vancomycin in a highly vancomycin-resistant *Streptomyces coelicolor* mutant. *Future Microbiol.* 9 603–622. 10.2217/fmb.14.21 24957088

[B87] Santos-Beneit F. Martín J. F. (2013). Vancomycin resistance in *Streptomyces coelicolor* is phosphate-dependent but is not mediated by the PhoP regulator. *J. Glob. Antimicrob. Resist.* 1 109–113. 10.1016/j.jgar.2013.03.003 27873577

[B88] Santos-Beneit F. Rodriguez-Garcia A. Sola-Landa A. Martin J. F. (2009). Crosstalk between two global regulators in *Streptomyces*: PhoP and AfsR interact in the control of *afsS*, *pstS* and *phoRP* transcription. *Mol. Microbiol.* 72 53–68. 10.1111/j.1365-2958.2009.06624.x 19220751

[B89] Sola-Landa A. Moura R. S. Martín J. F. (2003). The two-component PhoR-PhoP system controls both primary metabolism and secondary metabolite biosynthesis in *Streptomyces lividans*. *Proc. Natl. Acad. Sci. U.S.A.* 100 6133–6138. 10.1073/pnas.0931429100 12730372 PMC156338

[B90] Som N. F. Heine D. Holmes N. Knowles F. Chandra G. Seipke R. F. (2017). The MtrAB two-component system controls antibiotic production in *Streptomyces coelicolor* A3(2). *Microbiology* 163 1415–1419. 10.1099/mic.0.000524 28884676 PMC5845573

[B91] Stankovic N. Senerovic L. Ilic-Tomic T. Vasiljevic B. Nikodinovic-Runic J. (2014). Properties and applications of undecylprodigiosin and other bacterial prodigiosins. *Appl. Microbiol. Biotechnol.* 98 3841–3858. 10.1007/s00253-014-5590-1 24562326

[B92] Suntharalingam P. Senadheera M. D. Mair R. W. Levesque C. M. Cvitkovitch D. G. (2009). The LiaFSR system regulates the cell envelope stress response in *Streptococcus mutans*. *J. Bacteriol.* 191 2973–2984. 10.1128/jb.01563-08 19251860 PMC2681809

[B93] Tierney A. R. Rather P. N. (2019). Roles of two-component regulatory systems in antibiotic resistance. *Future Microbiol.* 14 533–552. 10.2217/fmb-2019-0002 31066586 PMC6526388

[B94] Tong Y. Charusanti P. Zhang L. Weber T. Lee S. Y. (2015). CRISPR-Cas9 based engineering of actinomycetal genomes. *ACS Synth. Biol.* 4 1020–1029. 10.1021/acssynbio.5b00038 25806970

[B95] van der Heul H. U. Bilyk B. L. McDowall K. J. Seipke R. F. van Wezel G. P. (2018). Regulation of antibiotic production in Actinobacteria: new perspectives from the post-genomic era. *Nat. Prod. Rep.* 35 575–604. 10.1039/c8np00012c 29721572

[B96] van der Meij A. Worsley S. F. Hutchings M. I. van Wezel G. P. (2017). Chemical ecology of antibiotic production by actinomycetes. *FEMS Microbiol. Rev.* 41 392–416. 10.1093/femsre/fux005 28521336

[B97] VanBogelen R. A. Neidhardt F. C. (1990). Ribosomes as sensors of heat and cold shock in *Escherichia coli*. *Proc. Natl. Acad. Sci. U.S.A.* 87 5589–5593. 10.1073/pnas.87.15.5589 2198567 PMC54372

[B98] Waterhouse A. Bertoni M. Bienert S. Studer G. Tauriello G. Gumienny R. (2018). SWISS-MODEL: homology modelling of protein structures and complexes. *Nucleic Acids Res.* 46 W296–W303. 10.1093/nar/gky427 29788355 PMC6030848

[B99] Xia H. Zhan X. Mao X. M. Li Y. Q. (2020). The regulatory cascades of antibiotic production in *Streptomyces*. *World J. Microbiol. Biotechnol.* 36:13. 10.1007/s11274-019-2789-4 31897764

[B100] Xu G. Wang J. Wang L. Tian X. Yang H. Fan K. (2010). “Pseudo” gamma-butyrolactone receptors respond to antibiotic signals to coordinate antibiotic biosynthesis. *J. Biol. Chem.* 285 27440–27448. 10.1074/jbc.M110.143081 20562102 PMC2930742

[B101] Yang S. J. Bayer A. S. Mishra N. N. Meehl M. Ledala N. Yeaman M. R. (2012). The *Staphylococcus aureus* two-component regulatory system, GraRS, senses and confers resistance to selected cationic antimicrobial peptides. *Infect. Immun.* 80 74–81. 10.1128/iai.05669-11 21986630 PMC3255649

[B102] Yeo K. J. Hong Y. S. Jee J. G. Lee J. K. Kim H. J. Park J. W. (2014). Mechanism of the pH-induced conformational change in the sensor domain of the DraK Histidine kinase via the E83, E105, and E107 residues. *PLoS One* 9:e107168. 10.1371/journal.pone.0107168 25203403 PMC4159317

[B103] Yepes A. Rico S. Rodríguez-García A. Santamaría R. I. Díaz M. (2011). Novel two-component systems implied in antibiotic production in *Streptomyces coelicolor*. *PLoS One* 6:e19980. 10.1371/journal.pone.0019980 21625497 PMC3098853

[B104] Yin S. Daum R. S. Boyle-Vavra S. (2006). VraSR two-component regulatory system and its role in induction of *pbp2* and *vraSR* expression by cell wall antimicrobials in *Staphylococcus aureus*. *Antimicrob. Agents Chemother.* 50 336–343. 10.1128/AAC.50.1.336-343.2006 16377706 PMC1346790

[B105] Zhang G. Tian Y. Hu K. Feng C. Tan H. (2010). SCO3900, co-transcripted with three downstream genes, is involved in the differentiation of *Streptomyces coelicolor*. *Curr. Microbiol.* 60 268–273. 10.1007/s00284-009-9536-2 20012957

[B106] Zhang G. Tian Y. Hu K. Zhu Y. Chater K. F. Feng C. (2012). Importance and regulation of inositol biosynthesis during growth and differentiation of *Streptomyces*. *Mol. Microbiol.* 83 1178–1194. 10.1111/j.1365-2958.2012.08000.x 22329904

