# Supplementary material for: Antibiotic Production and Antibiotic Resistance: The Two Sides of AbrB1/B2, a Two-Component System of Streptomyces coelicolor
Source: Front Microbiol. 2020 Oct 9;11:587750. doi: 10.3389/fmicb.2020.587750 (PMC7581861; doi:10.3389/fmicb.2020.587750)
Supplement: Supplementary file 1 [file Image_1.pdf]

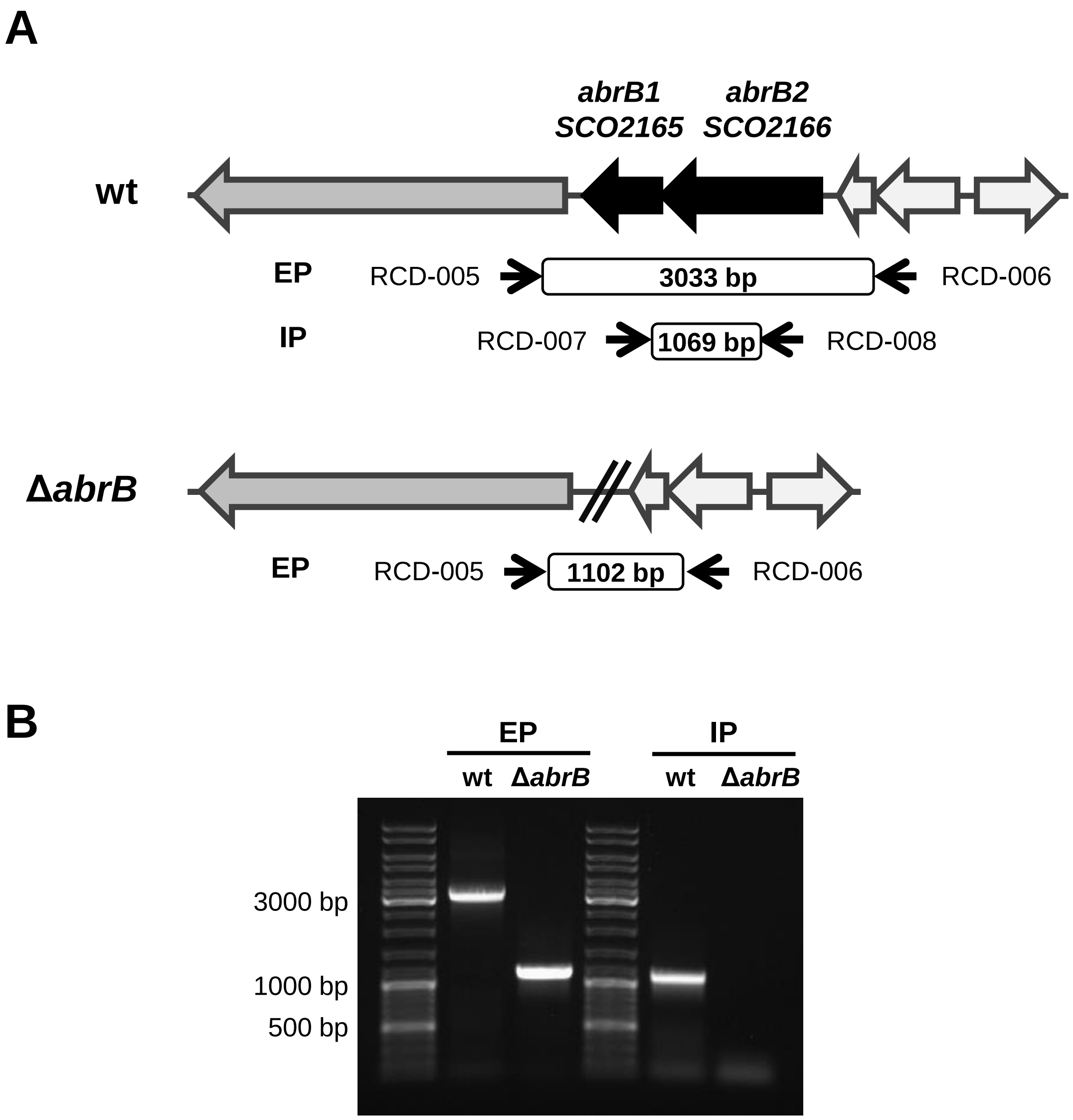

Figure S1. Checking of the *abrB1/B2* Deletion.

A) Image of the location of the external and internal primers (EP and IP respectively) in the genome of *S. coelicolor* M145 wt and mutant Δ*abrB* strains. The sizes of the fragments generated by PCR are indicated in each case.

B) PCR fragments obtained using the corresponding EP and IP primers in both strains.
