# Supplementary material for: Antibiotic Production and Antibiotic Resistance: The Two Sides of AbrB1/B2, a Two-Component System of Streptomyces coelicolor
Source: Front Microbiol. 2020 Oct 9;11:587750. doi: 10.3389/fmicb.2020.587750 (PMC7581861; doi:10.3389/fmicb.2020.587750)
Supplement: Supplementary file 2 [file Image_2.pdf]

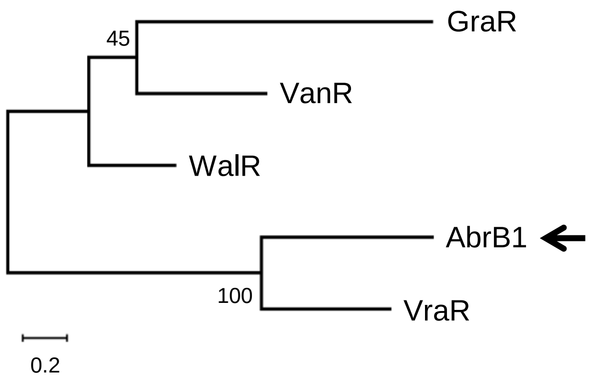

Figure S2. Maximum Likelihood Analysis of RRs. Maximum likelihood analysis of the following RRs: AbrB1 from *S. coelicolor* (pointed by the arrow), and VraR, VanR, WalR and GraR from *S. aureus* (all of them implied in vancomycin resistance). The tree with the highest log likelihood (-2758.14) is shown. The percentage of replicate trees in which the associated taxa clustered together in the bootstrap test (1000 replicates) are shown next to the branches. The tree is drawn to scale, with branch lengths measured in the number of substitutions per site. The analysis was conducted in *MEGA X*.
