# Supplementary material for: Antibiotic Production and Antibiotic Resistance: The Two Sides of AbrB1/B2, a Two-Component System of Streptomyces coelicolor
Source: Front Microbiol. 2020 Oct 9;11:587750. doi: 10.3389/fmicb.2020.587750 (PMC7581861; doi:10.3389/fmicb.2020.587750)
Supplement: Supplementary file 3 [file Image_3.pdf]

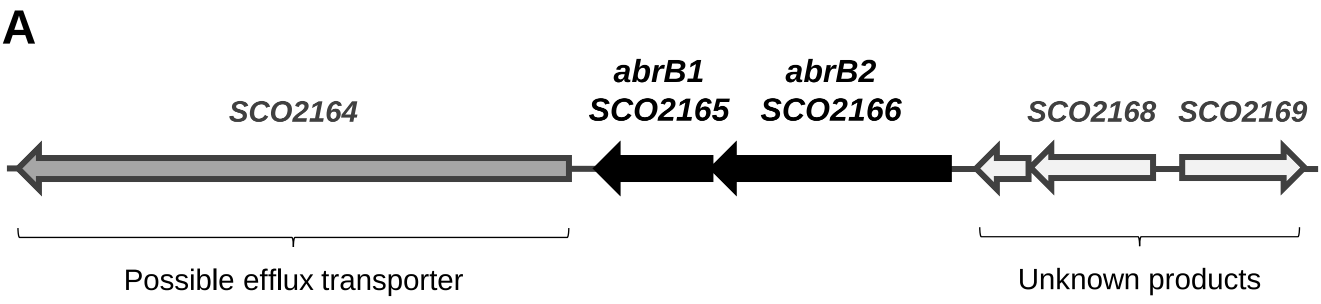

	Name	Product	Start	End	Strand	Lenght
SCO2165	*abrB1*	RR	2329632	2330315	-1	684 nt
SCO2166	*abrB2*	HK	2330312	2331562	-1	1251 nt

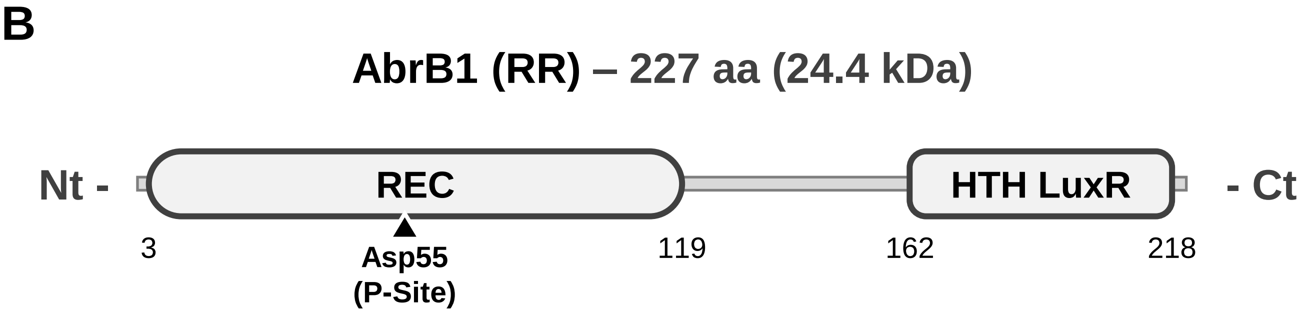

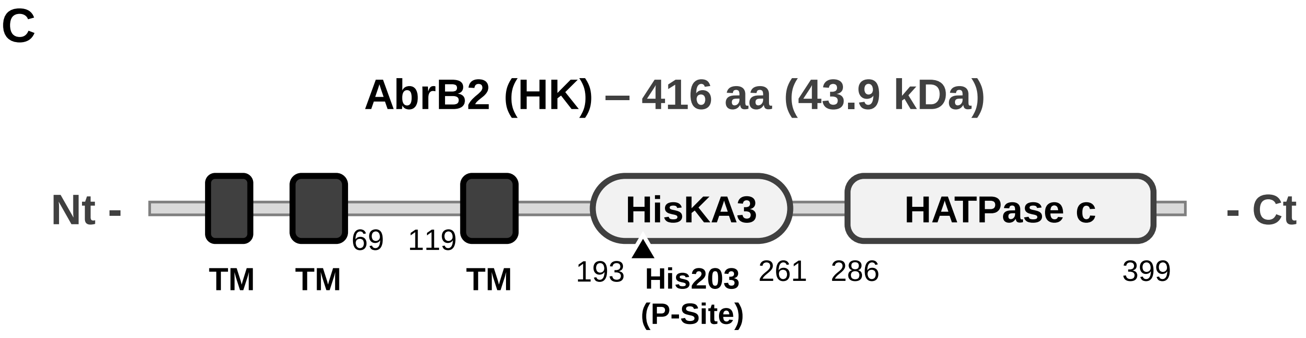

Figure S3. Overview of AbrB1/B2.

A) Genomic context and information of *abrB1/B2* (*SCO2165/SCO2166*).

B) Domain architecture of AbrB1 (RR). Phosphorylation site is indicated (P-site).

C) Domain architecture of AbrB2 (HK). Phosphorylation site is indicated (P-site).
