# Supplementary material for: Antibiotic Production and Antibiotic Resistance: The Two Sides of AbrB1/B2, a Two-Component System of Streptomyces coelicolor
Source: Front Microbiol. 2020 Oct 9;11:587750. doi: 10.3389/fmicb.2020.587750 (PMC7581861; doi:10.3389/fmicb.2020.587750)
Supplement: Supplementary file 4 [file Image_4.pdf]

***Streptomyces coelicolor***

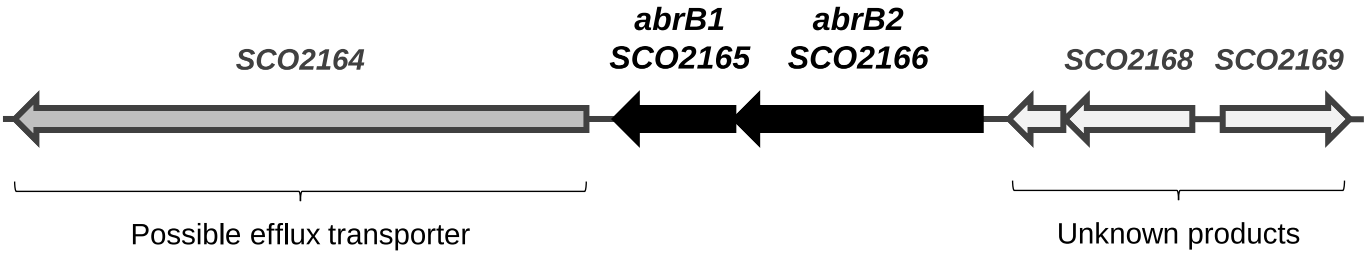


| Specie and Genomic Context | Amino Acid Sequence Identity (I) and Similarity (S): AbrB1 Homolog (RR) | Amino Acid Sequence Identity (I) and Similarity (S): AbrB2 Homolog (HK) |
|---|---|---|
| ***Streptomyces avermitilis*** 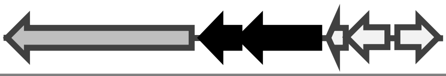 | **SAV_6039**<br>I: 91%<br>S: 96% | **SAV_6038**<br>I: 77%<br>S: 83% |
| ***Streptomyces hygroscopicus*** 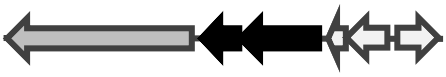 | **SHJG_3644**<br>I: 88%<br>S: 91% | **SHJG_3645**<br>I: 78%<br>S: 84% |
| ***Streptomyces scabiei*** 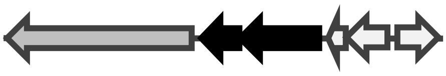 | **SCAB_67211**<br>I: 87%<br>S: 93% | **SCAB_67201**<br>I: 74%<br>S: 80% |
| ***Streptomyces albus*** (Inversion) 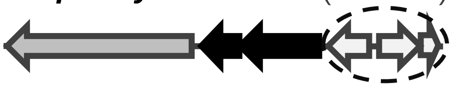 | **DUI70_5610**<br>I: 81%<br>S: 88% | **DUI70_5609**<br>I: 70%<br>S: 76% |
| ***Streptomyces venezuelae*** 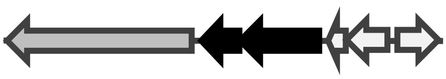 | **SVEN_1822**<br>I: 81%<br>S: 87% | **SVEN_1823**<br>I: 64%<br>S: 70% |
| ***Streptomyces griseus*** 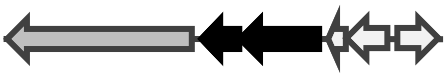 | **SGR_5344**<br>I: 77%<br>S: 82% | **SGR_5343**<br>I: 69%<br>S: 77% |

**Figure S4. Conservation of AbrB1/B2 in *Streptomyces* spp.**
Genomic context of *abrB1/B2* and its homologs in different *Streptomyces* species. Amino acid sequence identity (I) and similarity (S) to each homolog are indicated.
