# Supplementary material for: Antibiotic Production and Antibiotic Resistance: The Two Sides of AbrB1/B2, a Two-Component System of Streptomyces coelicolor
Source: Front Microbiol. 2020 Oct 9;11:587750. doi: 10.3389/fmicb.2020.587750 (PMC7581861; doi:10.3389/fmicb.2020.587750)
Supplement: Supplementary file 5 [file Image_5.pdf]

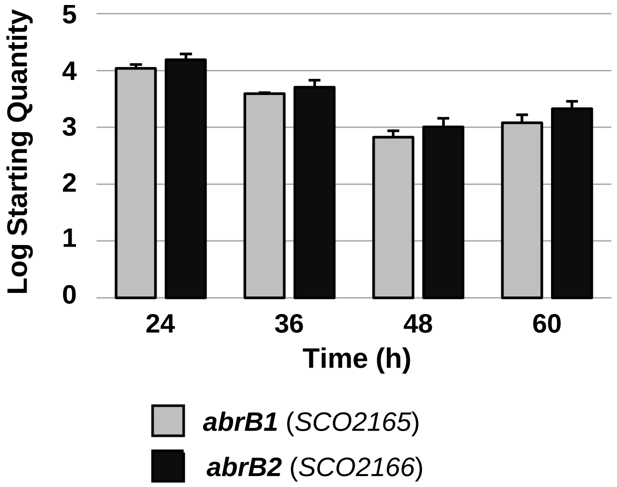

Figure S5. Expression Pattern of *abrB1/B2*. Time course expression by RT-qPCR of both genes of the AbrB1/B2 system: *abrB1* (*SCO2165*; grey color) and *abrB2* (*SCO2166*; black color) in the wt strain. Error bars show the standard deviation of the quadruplicate assays.
