# Supplementary material for: Antibiotic Production and Antibiotic Resistance: The Two Sides of AbrB1/B2, a Two-Component System of Streptomyces coelicolor
Source: Front Microbiol. 2020 Oct 9;11:587750. doi: 10.3389/fmicb.2020.587750 (PMC7581861; doi:10.3389/fmicb.2020.587750)
Supplement: Supplementary file 6 [file Image_6.pdf]

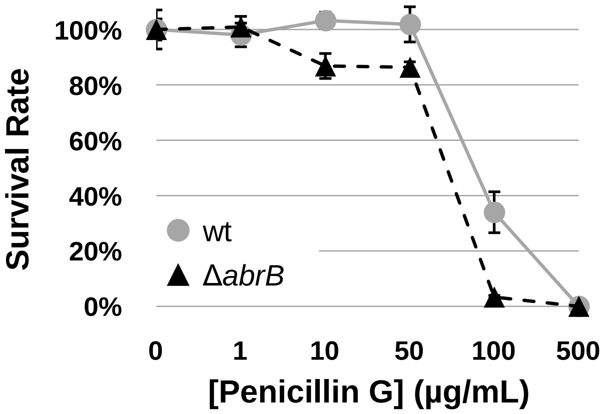

Figure S6. Penicillin G Survival Assay.
Survival assay of spores from *S. coelicolor* M145 (wt; grey circles) and mutant Δ*abrB* strains (black triangles) against different concentrations of penicillin G in NMMP plates. Error bars show the standard deviation of quadruplicate assays.
