# Supplementary material for: Antibiotic Production and Antibiotic Resistance: The Two Sides of AbrB1/B2, a Two-Component System of Streptomyces coelicolor
Source: Front Microbiol. 2020 Oct 9;11:587750. doi: 10.3389/fmicb.2020.587750 (PMC7581861; doi:10.3389/fmicb.2020.587750)
Supplement: Supplementary file 7 [file Table_1.pdf]

Table S1. Bacterial Strains Used in this Work.

Bacterial strain	Genotype	Reference
Escherichia coli DH5α	F-. ϕ80d*lacZ*ΔM15, Δ(*lacZYAargF*)U169, *recA1. endA1, hsdR17*(rk-. mk+), *supE44,* λ-, *thi-1*, *gyrA*. *relA1*	(Sambrook et al., 1989)
Escherichia coli ET12567	*Dam, dcm, hsdS, cat, tet*	(MacNeil et al., 1992)
Streptomyces coelicolor M145	SCP1- SCP2-	(Hopwood et al., 1985)
Streptomyces coelicolor M145 Δ*abrB*	*S. coelicolor* M145 derivative; Δ*SCO2165/66*	This work
Staphylococcus epidermidis ATCC 14990	Wild type	-
Enterococcus faecalis ATCC 29212	Wild type	-

References

Hopwood, D.A., Bibb, J.M., Chater, K.F., Kieser, T., Bruton, C.J., Kieser, H.M., et al. (1985). *Genetic manipulation of Streptomyces: A laboratory manual.* Norwich, UK: John Innes Foundation.

MacNeil, D.J., Gewain, K.M., Ruby, C.L., Dezeny, G., Gibbons, P.H., and MacNeil, T. (1992). Analysis of *Streptomyces avermitilis* genes required for avermectin biosynthesis utilizing a novel integration vector. *Gene* 111(1), 61-68. doi: doi: 10.1016/0378-1119(92)90603-M.

Sambrook, J., Fritsch, E., and Maniatis, T. (1989). *Molecular cloning: a laboratory manual.* Cold Spring Harbor, N. Y.: Cold Spring Harbor Laboratory.
