# Supplementary material for: Antibiotic Production and Antibiotic Resistance: The Two Sides of AbrB1/B2, a Two-Component System of Streptomyces coelicolor
Source: Front Microbiol. 2020 Oct 9;11:587750. doi: 10.3389/fmicb.2020.587750 (PMC7581861; doi:10.3389/fmicb.2020.587750)
Supplement: Supplementary file 8 [file Table_2.pdf]

Table S2. Plasmids Used in this Work.

Plasmid	Characteristics	Reference
pUZ8002	Plasmid to conjugative transfer (operon *tra*).<br>*Resistances*: Neomycin.	(Paget et al., 1999)
pCRISPR-Cas9	Bifunctional plasmid *Escherichia – Streptomyces*. This plasmid includes *cas9* gene from *Streptococcus pyogenes* under *tipA* promoter, and sgRNA cassette under *ermE* promoter. Thermosensitive.<br>*Resistances:* Apramycin, Thiostrepton.	(Tong et al., 2015)
pCRISPR-Cas9-sgB	pCRISPR-Cas9 derivative. sgRNA targeted to *abrB1/B2* system.	This work
pCRISPR-Cas9-AbrB	pCRISPR-Cas9-sgB derivative. This plasmid includes the homologous recombination template for adjacent sequences to a*brB1/B2* system genes.	This work
pN702GEM3	Bifunctional plasmid *Escherichia – Streptomyces*. High copy number.<br>*Resistances*: Neomycin.	(Fernández-Abalos et al., 2003)
pNBAbrB	pN702GEM3 derivative. This plasmid includes *abrB1/B2* operon under its own promoter.	This work
pXHis1	*Escherichia coli* plasmid. *Resistances:* Ampicillin	(Adham et al., 2001)
pXAbrB	pXHis1 derivative. This plasmid includes *abrB1/B2* operon under its own promoter.	This work
pKC796	Bifunctional plasmid *Escherichia – Streptomyces*. Integrative (contains phage φC31 integrase).<br>*Resistances:* Apramycin	(Kuhstoss et al., 1991)
pKCAbrB	pKC796 derivative. This plasmid includes *abrB1/B2* operon under its own promoter.	This work

References

Adham, S.A., Honrubia, P., Díaz, M., Fernández-Ábalos, J.M., Santamaría, R.I., and Gil, J.A. (2001). Expression of the genes coding for the xylanase Xys1 and the cellulase Cel1 from the straw-decomposing *Streptomyces halstedii* JM8 cloned into the amino-acid producer *Brevibacterium lactofermentum* ATCC13869. *Arch Microbiol* 177(1), 91-97.

Fernández-Abalos, J.M., Reviejo, V., Díaz, M., Rodríguez, S., Leal, F., and Santamaría, R.I. (2003). Posttranslational processing of the xylanase Xys1L from *Streptomyces halstedii* JM8 is carried out by secreted serine proteases. *Microbiology* 149, 1623-1632.

Kuhstoss, S., Richardson, M.A., and Rao, R.N. (1991). Plasmid cloning vectors that integrate site-specifically in *Streptomyces* spp. *Gene* 97(1), 143-146.

Paget, M.S., Leibovitz, E., and Buttner, M.J. (1999). A putative two-component signal transduction system regulates *sigmaE*, a sigma factor required for normal cell wall integrity in *Streptomyces coelicolor A3*(2). *Mol Microbiol* 33(1), 97-107.

Tong, Y., Charusanti, P., Zhang, L., Weber, T., and Lee, S.Y. (2015). CRISPR-Cas9 Based Engineering of Actinomycetal Genomes. *ACS Syn Biol*. doi: 10.1021/acssynbio.5b00038.
