# Supplementary material for: Antibiotic Production and Antibiotic Resistance: The Two Sides of AbrB1/B2, a Two-Component System of Streptomyces coelicolor
Source: Front Microbiol. 2020 Oct 9;11:587750. doi: 10.3389/fmicb.2020.587750 (PMC7581861; doi:10.3389/fmicb.2020.587750)
Supplement: Supplementary file 9 [file Table_3.pdf]

Table S3. Oligonucleotides Used in this Work.

Primer	Sequence (5´ - 3´)	Application
SAM-051	ACGCCTACGTAAAAAAAGCACCGACTC GGTGCC	Reverse primer for sgRNA cassette construction. SnaBI site is underlined.
SAM-056	TCCACACGTGGCACCGCGAT	Primer for sequencing of sgRNA cassettes.
SAM-057	AGAGCATCACCGGCCTGTAC	Primer for sequencing of homology templates inserted in XbaI site.
SAM-058	CTAACGTCTGGAAAGACGACA	Primer for sequencing of homology templates inserted in XbaI site.
SAM-067	CATGCCATGG***GCAGGACACGGATCGTC ATA***GTTTTAGAGCTAGAAATAGC	Forward primer for sgRNA AbrB cassette construction. Specific AbrB sequence in bold italic. NcoI site is underlined.
SAM-068	GCAGGACACGGATCGTCATA	Forward primer for verifying pCRISPR-Cas9-sgAbrB plasmid.
SAM-069	TTTTTTTCTAGAGGAGCTTGTCCTCGAC GGCG	Forward primer for building of a*brB* upstream homologous template for pCRISPR-Cas9. XbaI site is underlined.
SAM-070	CCGGCTACCGTAAAAGACCCCTCCGCC CGCGTGTACTGC	Reverse primer for building of a*brB* upstream homologous template for pCRISPR-Cas9.
SAM-071	CAGTACACGCGGGCGGAGGGGTCTTTT ACGGTAGCCGGA	Forward primer for building of a*brB* downstream homologous template for pCRISPR-Cas9.
SAM-072	TTTTTTTCTAGAGTCGGGCAAGCTGGAG GACC	Reverse primer for building of a*brB* downstream homologous template for pCRISPR-Cas9. XbaI site is underlined.
RCD-005	ATGATCTTCCGCGCGAAGGC	Forward external primer for verifying *ΔabrB* genomic deletion.
RCD-006	GGGTCAGGGTCTCTCTTCTG	Reverse external primer for verifying *ΔabrB* genomic deletion.
RCD-007	GAGGAAGCCGGAGGCCCCGGCCCGC	Forward internal primer for verifying *ΔabrB* genomic deletion.
RCD-008	GCCGCGCCTTCGTGCAGGCCATCCGGG	Reverse internal primer for verifying *ΔabrB* genomic deletion.
DSM-002	TATATAAAGCTTCACTCCACCCTTGGAC GGACC	Forward primer for amplify *abrB* operon under its own promoter. HindIII site is underlined.
DSM-004	TATATAGAATTCGGCGCCTTGCTTCGC	Reverse primer for amplify *abrB* operon under its own promoter. HindIII site is underlined.
RCD-009	GGACAAGGTCGAGAAGAAC	Forward primer for RT-qPCR of *rpsL* (*SCO4659*)
RCD-010	GAGTTCGGCTTCTTCGG	Reverse primer for RT-qPCR of *rpsL* (*SCO4659*)
RCD-015	CCTCGAAGGTGGTCAGTATG	Forward primer for RT-qPCR of *abrB1* (*SCO2165*)
RCD-016	ACGTGGTGCTGATGGAC	Reverse primer for RT-qPCR of *abrB1* (*SCO2165*)

RCD-017	CCTGGATGATGCGGTAGG	Forward primer for RT-qPCR of *abrB2* (*SCO2166*)
RCD-018	AACTCGTCGCCACCTTC	Reverse primer for RT-qPCR of *abrB2* (*SCO2166*)
RCD-031	GAGTCCTTCGACGAGATGC	Forward primer for RT-qPCR of *bfr* (*SCO2113*)
RCD-032	GAAGAGCCGCTGGTAGTTC	Reverse primer for RT-qPCR of *bfr* (*SCO2113*)
RCD-045	TCGTGATGATGCTGCAC	Forward primer for RT-qPCR of *vanJ* (*SCO3592*)
RCD-046	CGAACAGACCGAACCAC	Reverse primer for RT-qPCR of *vanJ* (*SCO3592*)
RCD-047	ATGCTGTCCGAGTCCTTC	Forward primer for RT-qPCR of *SCO7536*
RCD-048	CCAGTCCGGTGATCTTCTC	Reverse primer for RT-qPCR of *SCO7536*
RCD-049	TACGTGGCCCTCTACAC	Forward primer for RT-qPCR of *ecrA1* (*SCO2518*)
RCD-050	TGGGTGTCGACGATGAG	Reverse primer for RT-qPCR of *ecrA1* (*SCO2518*)
RCD-051	GTCATCACGTTCACCTTCC	Forward primer for RT-qPCR of *redG* (*SCO5897*)
RCD-052	GATGTGCTCGGTGTTCTG	Reverse primer for RT-qPCR of *redG* (*SCO5897*)
RCD-053	TTGTTGCTGCTCATCTCTC	Forward primer for RT-qPCR of *mfnB* (*SCO6440*)
RCD-054	TCGTCGGGCTTCTTGAC	Reverse primer for RT-qPCR of *mfnB* (*SCO6440*)
