# Supplementary material for: Antibiotic Production and Antibiotic Resistance: The Two Sides of AbrB1/B2, a Two-Component System of Streptomyces coelicolor
Source: Front Microbiol. 2020 Oct 9;11:587750. doi: 10.3389/fmicb.2020.587750 (PMC7581861; doi:10.3389/fmicb.2020.587750)
Supplement: Supplementary file 10 [file Table_4.pdf]

Table S4. **Differentially Expressed Genes at 24 h (RNA-Seq).**
Genes up-regulated (FC > 2; green shadowed) and down-regulated (FC < -2; magenta shadowed) in the mutant strain *S. coelicolor* M145 Δ*abrB* relative to wild type in NMMP at 24 hours. The indicated genes passed the filter padj ≤ 0.05.

Gene ID	Gene Name	FC	p-value	padj	Description	Reference
SCO1550	-	**-2.2**	4.5E-18	8.7E-15	Unknown protein	-
SCO2165	*abrB1*	**-14.1**	2.0E-14	1.7E-11	TCS Response Regulator	This work
SCO2166	*abrB2*	**-10.8**	1.7E-16	2.6E-13	TCS Histidine Kinase	This work
SCO3899	*inoA*	**-3.1**	3.5E-97	1.4E-93	Myo-Inositol-1-Phosphate Synthase	(Zhang et al., 2012)
SCO3900	*inoR*	**-2.8**	9.2E-39	2.4E-35	Transcriptional Regulator	(Zhang et al., 2012)
SCO4902	-	**2.4**	9.6E-10	3.1E-07	ATP Binding Protein	-
SCO6273	*cpkC*	**-2.0**	9.0E-04	2.9E-02	Type I Polyketide Synthase	(Pawlik et al., 2007)
SCO6274	*cpkB*	**-2.2**	6.8E-05	4.0E-03	Type I Polyketide Synthase	(Pawlik et al., 2007)
SCO6275	*cpkA*	**-2.3**	1.5E-05	1.0E-03	Type I Polyketide Synthase	(Pawlik et al., 2007)
SCO6682	*ramS*	**2.9**	8.1E-12	4.5E-09	Lanthionine-Containing Peptide SapB	(Kodani et al., 2004)
SCO6931	-	**2.3**	3.7E-07	4.5E-05	Unknown protein	-
SCO7013	-	**7.1**	7.9E-16	8.8E-13	Extracellular Solute-Binding Protein	-
SCO7014	-	**4.2**	1.5E-07	2.2E-05	Transcriptional Regulator	-
SCO7314	*sigM*	**2.1**	2.4E-09	6.6E-07	RNA Polymerase Sigma Factor	(Lee et al., 2005)
SCO7536	-	**-17.2**	6.2E-258	4.8E-254	Transporter	-

References

Kodani, S., Hudson, M.E., Durrant, M.C., Buttner, M.J., Nodwell, J.R., and Willey, J.M. (2004). The SapB morphogen is a lantibiotic-like peptide derived from the product of the developmental gene *ramS* in *Streptomyces coelicolor*. *Proc Nat Acad Sci USA* 101(31)**,** 11448-11453. doi: 10.1073/pnas.0404220101.

Lee, E.J., Karoonuthaisiri, N., Kim, H.S., Park, J.H., Cha, C.J., Kao, C.M., et al. (2005). A master regulator sigmaB governs osmotic and oxidative response as well as differentiation via a network of sigma factors in *Streptomyces coelicolor*. *Mol Microbiol* 57(5)**,** 1252-1264. doi: 10.1111/j.1365-2958.2005.04761.x.

Pawlik, K., Kotowska, M., Chater, K.F., Kuczek, K., and Takano, E. (2007). A cryptic type I polyketide synthase (*cpk*) gene cluster in *Streptomyces coelicolor A3(2)*. *Arch Microbiol* 187(2)**,** 87-99.

Zhang, G., Tian, Y., Hu, K., Zhu, Y., Chater, K.F., Feng, C., et al. (2012). Importance and regulation of inositol biosynthesis during growth and differentiation of *Streptomyces*. *Mol Microbiol* 83(6)**,** 1178-1194. doi: 10.1111/j.1365-2958.2012.08000.x.
