# Supplementary material for: Antibiotic Production and Antibiotic Resistance: The Two Sides of AbrB1/B2, a Two-Component System of Streptomyces coelicolor
Source: Front Microbiol. 2020 Oct 9;11:587750. doi: 10.3389/fmicb.2020.587750 (PMC7581861; doi:10.3389/fmicb.2020.587750)
Supplement: Supplementary file 11 [file Table_5.pdf]

Table S5. Differentially Expressed Genes at 36 h (RNA-Seq).

Genes up-regulated (FC > 2; green shadowed) and down-regulated (FC < -2; magenta shadowed) in the mutant strain *S. coelicolor* M145 Δ*abrB* relative to wild type in NMMP at 36 hours. The indicated genes passed the filter padj ≤ 0.05.

Gene ID	Gene Name	FC	p-value	padj	Description	Reference
SCO0644	-	**-2.1**	4.2E-05	3.0E-03	Unknown protein	-
SCO1105	-	**2.5**	5.5E-08	1.5E-05	Unknown protein	-
SCO1294	-	**2.4**	2.4E-04	1.1E-02	Methionine Gamma-Lyase	-
SCO1528	-	**2.9**	4.7E-04	1.9E-02	Elongation Factor G	-
SCO1550	-	**-2.2**	4.1E-05	3.0E-03	Unknown protein	-
SCO1573	-	**-2.1**	7.6E-04	2.8E-02	Oxidoreductase	-
SCO1611	-	**2.2**	9.3E-08	2.1E-05	Dehydrogenase	(Krysenko et al., 2019)
SCO1612	-	**2.3**	1.5E-06	2.2E-04	Dehydrogenase	(Krysenko et al., 2019)
SCO1613	*glnA4*	**2.3**	8.1E-07	1.3E-04	Glutamine Synthetase	(Krysenko et al., 2019)
SCO1700	-	**-2.0**	6.6E-04	2.5E-02	Unknown protein	-
SCO1705	-	**2.2**	8.1E-04	2.8E-02	Oxidase	-
SCO1706	-	**2.9**	3.3E-05	3.0E-03	Dehydrogenase	-
SCO1746	-	**3.0**	3.4E-07	6.0E-05	Protease	-
SCO2113	*bfr*	**-2.9**	1.5E-04	8.0E-03	Bacterioferritin	-
SCO2165	*abrB1*	**-18.8**	1.8E-13	1.3E-10	TCS Response Regulator	This work
SCO2166	*abrB2*	**-39.6**	2.0E-20	3.9E-17	TCS Histidine Kinase	This work
SCO2275	-	**2.1**	5.3E-04	2.1E-02	Iron Transporter	-
SCO2360	-	**-4.0**	1.8E-05	2.0E-03	Unknown protein	-
SCO2494	-	**4.0**	1.5E-05	1.0E-03	Pyruvate Phosphate Dikinase	(Llamas-Ramírez et al., 2020)
SCO2517	*ecrA2*	**2.4**	2.0E-03	4.9E-02	TCS Response Regulator	(Li et al., 2004)
SCO2518	*ecrA1*	**2.5**	5.3E-04	2.1E-02	TCS Histidine Kinase	(Li et al., 2004)

SCO2519	-	**4.7**	7.9E-05	5.0E-03	Transporter	-
SCO2616	-	**3.0**	1.5E-05	1.0E-03	Unknown protein	-
SCO2722	-	**-5.3**	5.2E-16	5.6E-13	Transporter	-
SCO2723	-	**-6.6**	2.4E-23	9.3E-20	Transporter	-
SCO2783	*desB*	**-2.1**	1.5E-04	8.0E-03	Monooxygenase	(Barona-Gómez et al., 2004)
SCO2785	*desD*	**-2.1**	1.6E-04	8.0E-03	Siderophore Synthetase	(Barona-Gómez et al., 2004)
SCO2967	-	**-2.1**	2.0E-04	1.0E-02	Protease	-
SCO2979	*aglF*	**2.3**	1.5E-04	8.0E-03	Sugar Transporter	(Bertram et al., 2004)
SCO2980	*aglG*	**2.3**	1.4E-04	8.0E-03	Sugar Transporter	(Bertram et al., 2004)
SCO3589	*vanS*	**-2.2**	1.8E-05	2.0E-03	TCS Histidine Kinase	(Hong et al., 2004)
SCO3590	*vanR*	**-2.5**	9.1E-06	9.4E-04	TCS Response Regulator	(Hong et al., 2004)
SCO3592	*vanJ*	**-3.9**	1.1E-04	6.0E-03	Unknown protein	(Hong et al., 2004)
SCO3593	*vanK*	**-4.2**	1.7E-06	2.4E-04	Transferase	(Hong et al., 2004)
SCO3594	*vanH*	**-4.0**	1.6E-06	2.4E-04	D-Lactate Dehydrogenase	(Hong et al., 2004)
SCO3595	*vanA*	**-4.3**	2.3E-07	4.5E-05	D-Alanine: D-Lactate Ligase	(Hong et al., 2004)
SCO3596	*vanX*	**-3.6**	1.3E-04	7.0E-03	D-Alanine: D-Alanine Dipeptidase	(Hong et al., 2004)
SCO3657	-	**2.9**	5.3E-08	1.5E-05	Unknown protein	-
SCO3731	*scoF1*	**-2.3**	8.3E-05	5.0E-03	Cold-Shock Protein	(Kormanec and Sevcikova, 2000)
SCO4002	*nepA*	**2.5**	1.2E-04	7.0E-03	Structural Protein	(de Jong et al., 2009)
SCO4011	-	**-2.5**	2.4E-08	7.7E-06	Unknown protein	-
SCO4043	-	**-2.0**	7.4E-04	2.7E-02	Unknown protein	-
SCO4189	-	**3.0**	2.2E-05	2.0E-03	Unknown protein	-
SCO4214	-	**-2.2**	5.2E-04	2.1E-02	Unknown protein	-
SCO4224	-	**-2.3**	2.0E-04	1.0E-02	Lanthionine Synthetase	(Darbon et al., 2012)
SCO4979	*pckG*	**3.7**	1.5E-04	8.0E-03	Phosphoenolpyruvate Carboxykinase	(Llamas-Ramírez et al., 2020)
SCO5031	*ahpD*	**-3.1**	1.2E-05	1.0E-03	Alkyl Hydroperoxide Reductase	(Hahn et al., 2002)
SCO5142	-	**-2.2**	1.0E-03	3.9E-02	Unknown protein	-

SCO5207	-	**4.2**	2.5E-06	3.2E-04	Unknown protein	-
SCO5232	*dasA*	**-2.4**	1.3E-04	7.0E-03	Sugar Transporter	(Saito et al., 2007)
SCO5249	-	**3.1**	1.4E-09	7.4E-07	Nucleotide Binding Protein	-
SCO5250	-	**2.3**	9.0E-04	3.1E-02	Polyprenyl Synthetase	-
SCO5402	-	**-3.4**	9.8E-06	1.0E-03	Protease	-
SCO5403	*rapA1*	**-3.3**	3.1E-06	3.6E-04	TCS Response Regulator	(Lu et al., 2007)
SCO5404	*rapA2*	**-3.0**	1.1E-05	1.0E-03	TCS Histidine Kinase	(Lu et al., 2007)
SCO5447	-	**5.5**	3.2E-15	2.7E-12	Protease	(Kim et al., 2008)
SCO5575	-	**-2.0**	1.0E-03	4.2E-02	Unknown protein	-
SCO5883	*redU*	**2.5**	1.0E-03	3.9E-02	Unknown protein	-
SCO5884	-	**3.3**	1.4E-08	5.1E-06	Unknown protein	-
SCO5885	-	**3.9**	1.5E-14	1.1E-11	Unknown protein	-
SCO5888	*redP*	**3.4**	3.3E-06	3.7E-04	Reductase	(Cerdeño et al., 2001)
SCO5889	*redO*	**3.7**	1.1E-06	1.6E-04	Unknown protein	(Cerdeño et al., 2001)
SCO5890	*redN*	**3.0**	2.8E-07	5.3E-05	Synthase	(Cerdeño et al., 2001)
SCO5891	*redM*	**3.0**	5.9E-08	1.5E-05	Synthase	(Cerdeño et al., 2001)
SCO5892	*redL*	**2.8**	2.7E-08	8.4E-06	Synthase	(Cerdeño et al., 2001)
SCO5893	*redK*	**3.3**	6.6E-09	2.7E-06	Oxidoreductase	(Cerdeño et al., 2001)
SCO5894	*redJ*	**3.1**	1.1E-07	2.3E-05	Thioesterase	(Cerdeño et al., 2001)
SCO5895	*redI*	**3.5**	2.5E-09	1.1E-06	Methyltransferase	(Cerdeño et al., 2001)
SCO5896	*redH*	**3.2**	3.1E-10	1.7E-07	Synthetase	(Cerdeño et al., 2001)
SCO5897	*redG*	**2.5**	2.5E-05	2.0E-03	Oxidase	(Cerdeño et al., 2001)
SCO6026	*fadB1*	**2.4**	5.8E-04	2.3E-02	Fatty Acid Oxidation Complex	(Menéndez-Bravo et al., 2017)
SCO6027	*fadA1*	**2.6**	1.6E-04	8.0E-03	Thiolase	(Menéndez-Bravo et al., 2017)
SCO6045	-	**2.7**	1.9E-07	3.8E-05	Unknown protein	-
SCO6282	*cpkI*	**2.8**	8.9E-05	6.0E-03	Reductase	(Pawlik et al., 2007)
SCO6286	*scbr2*	**2.1**	8.0E-05	5.0E-03	Regulatory Protein	(Xu et al., 2010)

SCO6414	-	**3.2**	1.4E-07	3.0E-05	Ureidopropionase	-
SCO6415	*hyuA*	**3.3**	1.6E-09	8.0E-07	Hydantoinase	-
SCO6416	-	**3.1**	4.6E-08	1.3E-05	Dehydrogenase	-
SCO6440	*mfnB*	**9.9**	5.8E-16	5.6E-13	Synthase	-
SCO6441	-	**9.8**	1.4E-17	2.2E-14	Dehydrogenase	-
SCO6442	-	**9.4**	2.1E-16	2.7E-13	Dehydrogenase	-
SCO6443	-	**9.4**	6.9E-13	4.2E-10	Methyltransferase	-
SCO6543	-	**4.0**	5.0E-05	4.0E-03	Unknown protein	-
SCO6728	-	**-2.6**	4.5E-04	1.9E-02	Unknown protein	-
SCO7229	-	**-2.2**	1.0E-03	3.9E-02	Unknown protein	-
SCO7460	-	**-2.3**	4.0E-07	6.8E-05	Unknown protein	-
SCO7536	-	**-26.6**	1.5E-55	1.2E-51	Transporter	-
SCO7612	-	**-2.4**	8.7E-08	2.1E-05	Unknown protein	-

References

Barona-Gómez, F., Wong, U., Giannakopulos, A.E., Derrick, P.J., and Challis, G.L. (2004). Identification of a cluster of genes that directs desferrioxamine biosynthesis in *Streptomyces coelicolor* M145. *J Am Chem Soc* 126(50)**,** 16282-16283. doi: 10.1021/ja045774k.

Bertram, R., Schlicht, M., Mahr, K., Nothaft, H., Saier, M.H., Jr., and Titgemeyer, F. (2004). In silico and transcriptional analysis of carbohydrate uptake systems of *Streptomyces coelicolor* A3(2). *J Bacteriol* 186(5)**,** 1362-1373. doi: 10.1128/jb.186.5.1362-1373.2004.

Cerdeño, A.M., Bibb, M.J., and Challis, G.L. (2001). Analysis of the prodiginine biosynthesis gene cluster of *Streptomyces coelicolor* A3(2): new mechanisms for chain initiation and termination in modular multienzymes. *Chem Biol* 8(8)**,** 817-829. doi: 10.1016/s1074-5521(01)00054-0.

Darbon, E., Martel, C., Nowacka, A., Pegot, S., Moreau, P.L., and Virolle, M.J. (2012). Transcriptional and preliminary functional analysis of the six genes located in divergence of *phoR/phoP* in *Streptomyces lividans*. *Appl Microbiol Biotechnol* 95(6)**,** 1553-1566. doi: 10.1007/s00253-012-3995-2.

de Jong, W., Manteca, A., Sanchez, J., Bucca, G., Smith, C.P., Dijkhuizen, L., et al. (2009). NepA is a structural cell wall protein involved in maintenance of spore dormancy in *Streptomyces coelicolor*. *Mol Microbiol* 71(6)**,** 1591-1603.

Hahn, J.S., Oh, S.Y., and Roe, J.H. (2002). Role of OxyR as a peroxide-sensing positive regulator in *Streptomyces coelicolor* A3(2). *J Bacteriol* 184(19)**,** 5214-5222.

Hong, H.J., Hutchings, M.I., Neu, J.M., Wright, G.D., Paget, M.S., and Buttner, M.J. (2004). Characterization of an inducible vancomycin resistance system in *Streptomyces coelicolor* reveals a novel gene *(vanK)* required for drug resistance. *Mol Microbiol* 52(4)**,** 1107-1121.

Kim, D.W., Hesketh, A., Kim, E.S., Song, J.Y., Lee, D.H., Kim, I.S., et al. (2008). Complex extracellular interactions of proteases and a protease inhibitor influence multicellular development of *Streptomyces coelicolor*. *Mol Microbiol* 70(5)**,** 1180-1193. doi: 10.1111/j.1365-2958.2008.06471.x.

Kormanec, J., and Sevcikova, B. (2000). Identification and transcriptional analysis of a cold shock-inducible gene, *cspA*, in *Streptomyces coelicolor* A3(2). *Mol Gen Genet* 264(3)**,** 251-256. doi: 10.1007/s004380000298.

Krysenko, S., Matthews, A., Okoniewski, N., Kulik, A., Girbas, M.G., Tsypik, O., et al. (2019). Initial Metabolic Step of a Novel Ethanolamine Utilization Pathway and Its Regulation in *Streptomyces coelicolor* M145. *mBio* 10(3). doi: 10.1128/mBio.00326-19.

Li, Y.Q., Chen, P.L., Chen, S.F., Wu, D., and Zheng, J. (2004). A pair of two-component regulatory genes *ecrA1/A2* in *S. coelicolor*. *J Zhejiang Univ Sci* 5(2)**,** 173-179.

Llamas-Ramírez, R., Takahashi-Iniguez, T., and Flores, M.E. (2020). The phosphoenolpyruvate-pyruvate-oxaloacetate node genes and enzymes in *Streptomyces coelicolor* M-145. *Int Microbiol*. doi: 10.1007/s10123-019-00116-x.

Lu, Y., Wang, W., Shu, D., Zhang, W., Chen, L., Qin, Z., et al. (2007). Characterization of a novel two-component regulatory system involved in the regulation of both actinorhodin and a type I polyketide in *Streptomyces coelicolor*. *Appl Microbiol Biotechnol* 77(3)**,** 625-635.

Menéndez-Bravo, S., Paganini, J., Avignone-Rossa, C., Gramajo, H., and Arabolaza, A. (2017). Identification of FadAB Complexes Involved in Fatty Acid beta-Oxidation in *Streptomyces coelicolor* and Construction of a Triacylglycerol Overproducing strain. *Front Microbiol* 8**,** 1428. doi: 10.3389/fmicb.2017.01428.

Pawlik, K., Kotowska, M., Chater, K.F., Kuczek, K., and Takano, E. (2007). A cryptic type I polyketide synthase (*cpk*) gene cluster in *Streptomyces coelicolor A3(2)*. *Arch Microbiol* 187(2)**,** 87-99.

Saito, A., Shinya, T., Miyamoto, K., Yokoyama, T., Kaku, H., Minami, E., et al. (2007). The dasABC gene cluster, adjacent to dasR, encodes a novel ABC transporter for the uptake of N,N'-diacetylchitobiose in Streptomyces coelicolor A3(2). *Appl Environ Microbiol* 73(9)**,** 3000-3008.

Xu, G., Wang, J., Wang, L., Tian, X., Yang, H., Fan, K., et al. (2010). "Pseudo" gamma-butyrolactone receptors respond to antibiotic signals to coordinate antibiotic biosynthesis. *J Biol Chem* 285(35)**,** 27440-27448. doi: 10.1074/jbc.M110.143081.
