# Supplementary material for: Antibiotic Production and Antibiotic Resistance: The Two Sides of AbrB1/B2, a Two-Component System of Streptomyces coelicolor
Source: Front Microbiol. 2020 Oct 9;11:587750. doi: 10.3389/fmicb.2020.587750 (PMC7581861; doi:10.3389/fmicb.2020.587750)
Supplement: Supplementary file 12 [file Table_6.pdf]

Table S6. **Other Relevant Genes (RNA-Seq).**

Genes related to actinorhodin production, up-regulated (FC > 2) in the mutant strain *S. coelicolor* M145 Δ*abrB* relative to wild type in NMMP at 36 hours. These genes did not pass the padj filter by little (0.05 < padj < 0.08).

Gene ID	Gene Name	FC	p-value	padj	Description	Reference
SCO5089	*actI-ORF3*	**5.0**	2.0E-03	5.5E-02	Polyketide synthase ACP	(Fernández-Moreno et al., 1992)
SCO5090	*actVII*	**4.3**	3.5E-03	8.0E-02	Cyclase/Dehydratase	(Fernández-Moreno et al., 1992)
SCO5091	*actIV*	**4.5**	2.4E-03	6.2E-02	Cyclase	(Fernández-Moreno et al., 1992)
SCO5092	*actVB*	**4.7**	2.1E-03	5.8E-02	Poliyketide putative dimerase	(Fernández-Moreno et al., 1992)

References

Fernández-Moreno, M.A., Martínez, E., Boto, L., Hopwood, D.A., and Malpartida, F. (1992). Nucleotide sequence and deduced functions of a set of cotranscribed genes of *Streptomyces coelicolor* A3(2) including the polyketide synthase for the antibiotic actinorhodin. *J Biol Chem* 267(27), 19278-19290.
