# Supplementary material for: Antibiotic Production and Antibiotic Resistance: The Two Sides of AbrB1/B2, a Two-Component System of Streptomyces coelicolor
Source: Front Microbiol. 2020 Oct 9;11:587750. doi: 10.3389/fmicb.2020.587750 (PMC7581861; doi:10.3389/fmicb.2020.587750)
Supplement: Supplementary file 13 [file Table_7.pdf]

Table S7. Validation of RNA-Seq Data by RT-qPCR.

Comparison of fold-changes (FC) of several genes between RNA-Seq data and RT-qPCR data. The RT-qPCRs controls employed were the housekeeping gene *rpsL* (*SCO4659*) and *abrB2* (internal control).

Gene	FC (RNA-Seq)	FC (RT-qPCR)
bfr (*SCO2113*)	-2.9	-3.1
vanJ (*SCO3592*)	-3.9	-3.2
SCO7536	-26.6	-21.5
ecrA1 (*SCO2518*)	2.5	7.1
redG (*SCO5897*)	2.5	5.9
mfnB (*SCO6440*)	9.9	22.5
