# Supplementary material for: Antibiotic Production and Antibiotic Resistance: The Two Sides of AbrB1/B2, a Two-Component System of Streptomyces coelicolor
Source: Front Microbiol. 2020 Oct 9;11:587750. doi: 10.3389/fmicb.2020.587750 (PMC7581861; doi:10.3389/fmicb.2020.587750)
Supplement: Supplementary file 14 [file Table_8.pdf]

Table S8. Homologs Search for the RR AbrB1.

Amino acid sequence comparisons between different RRs. Pairwise alignments were performed in *EMBOSS Needle* (EMBL-EBI), which uses the Needleman-Wunsch algorithm, applying a BLOSUM62 substitution matrix.

Protein	Organism	Identity	Similarity
AbrB1<br>VraR	*Streptomyces coelicolor*<br>*Staphylococcus aureus*	34 %	55 %
VanR<br>VanR	*Streptomyces coelicolor*<br>*Staphylococcus aureus*	31 %	48 %
AbrB1<br>LiaR	*Streptomyces coelicolor*<br>*Enterococcus faecium*	35 %	54 %
